# Recent Advances in Self-Powered Sensors Based on Ionic Hydrogels

**DOI:** 10.34133/research.0571

**Published:** 2025-01-14

**Authors:** Jianyu Yin, Peixue Jia, Ziqi Ren, Qixiang Zhang, Wenzhong Lu, Qianqian Yao, Mingfang Deng, Xubin Zhou, Yihua Gao, Nishuang Liu

**Affiliations:** School of Physics & Wuhan National Laboratory for Optoelectronics (WNLO), Huazhong University of Science and Technology (HUST), Wuhan 430074, China.

## Abstract

After years of research and development, flexible sensors are gradually evolving from the traditional “electronic” paradigm to the “ionic” dimension. Smart flexible sensors derived from the concept of ion transport are gradually emerging in the flexible electronics. In particular, ionic hydrogels have increasingly become the focus of research on flexible sensors as a result of their tunable conductivity, flexibility, biocompatibility, and self-healable capabilities. Nevertheless, the majority of existing sensors based on ionic hydrogels still mainly rely on external power sources, which greatly restrict the dexterity and convenience of their applications. Advances in energy harvesting technologies offer substantial potential toward engineering self-powered sensors. This article reviews in detail the self-powered mechanisms of ionic hydrogel self-powered sensors (IHSSs), including piezoelectric, triboelectric, ionic diode, moist-electric, thermoelectric, potentiometric transduction, and hybrid modes. At the same time, structural engineering related to device and material characteristics is discussed. Additionally, the relevant applications of IHSS toward wearable electronics, human–machine interaction, environmental monitoring, and medical diagnostics are further reviewed. Lastly, the challenges and prospective advancement of IHSS are outlined.

## Introduction

Over the past few years, the evolution of flexible electronics has continued to create innovative opportunities for “smart electronic skins” and wearable electronic devices [1–4]. As the central component of flexible electronic systems, flexible sensors can effectively transform various stimuli or physiological parameters into electronic signals [5–7]. This emerging field, spurred by the rapid development of electronic skin, has garnered increasing attention and research interest. As the demand for intelligence, portability, and comfort grows, the application prospects of flexible sensors have become even broader. In view of the increasing pursuit of intelligence, portability, and comfort, the application potential of flexible sensors is continuously expanding to many fields, including medical healthcare, motion monitoring, and environmental detection [8–10].

Although substantial advances in electronic sensors consisting of electronic components including conductors, semiconductors, and dielectrics have been achieved, there remains a technical challenge posed by the intimate communication between these devices and biological systems. This is because biological signaling is mediated by ions and molecules rather than electrons [11]. The introduction of ionic sensing has brought new vitality to bionic soft electronics. Since ionic devices adopt the same ion conduction as biological systems, ionic sensors can not only reconstruct the sensing topological structures of human skin but also simulate the sensing mechanisms that depend on ion migration under external stimuli such as pressure, strain, temperature, and humidity. Thus, compared with conventional electronic sensors, sensors based on ion transport have considerable conceptual similarities with biological systems [12]. Relying on the similar mechanism of skin perception, ionic sensors can respond to external stimuli by migration and redistribution of ions. Compared with conventional electron-mediated devices, this similarity ensures that ionic devices are able to mimic more advanced biocompatible interfaces and intelligent human–computer interactions, providing an effective tool and methodology for narrowing the gap between conventional electronics and biological interfaces.

Recently, ionic hydrogel possessing favorable ductility and adjustable conductivity, as a new type of conductive material, has been extensively applied in flexible sensors [13,14]. A very simple method for preparing ionic hydrogels is to directly introduce soluble inorganic salts or polyelectrolytes into the hydrogels [15]. Human skin functions like a flexible sensor, capable of accurately sensing various external stimuli through ion transport signals [16–18]. It also has advantages such as regenerability, self-healing capability, and good mechanical toughness, making it a subject of ongoing exploration and research for scientists [19–21]. As a soft material with high conductivity, ionic hydrogel’s 3-dimensional (3D) network structure offers unobstructed pathways for ion transport, enabling them to adapt to various complex deformations while maintaining stable electrical signal transmission [22,23]. Additionally, ionic hydrogels have mechanical properties and material compositions similar to biological tissues and exhibit good biocompatibility [24,25], indicating that they are expected to become widely used as the next generation of conductors in flexible sensor. These characteristics make ionic hydrogels critical in promoting the future development of flexible sensor technology, bringing new prospects for innovation and progress.

At present, many ionic hydrogel sensors follow the principle of resistance or capacitance [26–29], and their signal transmission carriers differ from external circuits, typically requiring operation with an external AC circuit. Consequently, to apply these ionic hydrogel-based sensors in flexible wearable devices, an external commercial battery or even a DC-AC conversion circuit is often necessary. However, this requirement limits the miniaturization, lightness, and wearable comfort of the sensor [30,31]. Additionally, commercial DC power sources can trigger electrochemical reactions on the electrode surface, affecting their chemical stability and sensing reliability [32]. Furthermore, traditional batteries, due to their short lifespan or need for periodic recharging, restrict the usage range of devices. Most batteries also contain toxic chemicals, posing environmental risks [33]. To address the issues caused by external power sources, self-powered ionic hydrogel-based sensors have emerged. In the backdrop of accelerated evolution of global economy and technologies, conventional energy supplies are experiencing increasing shortages. Hence, the exploitation and utilization of renewal energy sources has evolved into a central task that should be addressed for the sustainable development of modern society. Emerging energy harvesting technologies offer effective pathways for developing and utilizing new energy sources. In recent years, researchers have continued to explore diversified energy harvesting strategies, hoping to develop ionic hydrogel self-powered sensors (IHSSs) that eliminate the need for the external power supply. The energy conversion mechanisms include piezoelectric, triboelectric, ionic diode, moist-electric, thermoelectric, or potentiometric transduction. IHSS can efficiently capture mechanical, thermal, or moisture energy from environmental or biological movement and transform them to electricity, followed by an output as an electrical signal [34–39]. Consequently, IHSSs possess advantages such as flexibility, environmental sustainability, energy self-sufficiency, low cost, ease of preparation, and portability, making them widely applicable in smart wearables, healthcare, and environmental detection [40–43]. Traditional self-powered sensor devices based on inorganic materials are always stiff and incompatible with biological tissues, hindering the interaction between humans and wearable devices [44]. Also, rigid self-powered sensors can cause discomfort for the wearer and hinder the development of wearable and implantable electronics [45]. Furthermore, conventional self-powered sensors based on polymer elastomers are poorly biocompatible and they are not the preferred choice for use in the human body. They are limited by poor self-healing, limited degradation, and weak reversible self-adhesion in practical application scenarios [46]. Besides, hydrogel self-powered sensors based on electronic conductors are susceptible to deterioration of the conductive network [47]. Thus, in terms of flexibility, stretchability, biocompatibility, self-healing, degradability, reversible self-adhesion, and tissue similarity, ionic hydrogels based on ionic transport are becoming increasingly popular in the fabrication of wearable sensors attached to human skin as well as implantable sensors, and they are emerging as promising alternatives for the fabrication of the next generation of flexible sensors. Unlike traditional sensors, the core characteristic of IHSS devices is ion transport within the device in response to external stimuli. This unique mechanism endows IHSS devices with various design possibilities and a broad range of applications.

**Fig. 1.**
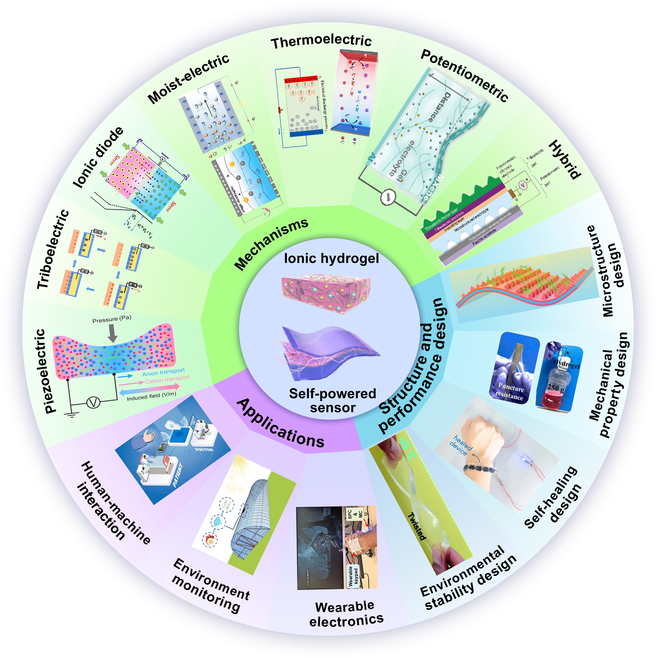
Overview of mechanisms, structure, and performance design applications of IHSS. Piezoelectric: Reproduced with permission from [58]. Copyright 2022, Science. Triboelectric: Reproduced with permission from [73]. Copyright 2023, Elsevier. Ionic diode: Reproduced with permission from [83]. Copyright 2023, Wiley-VCH. Moist-electric: Reproduced with permission from [39]. Copyright 2023, Wiley-VCH. Reproduced with permission from [99]. Copyright 2023, Wiley-VCH. Thermoelectric: Reproduced with permission from [114]. Copyright 2021, Elsevier. Reproduced with permission from [116]. Copyright 2024, Springer Nature. Potentiometric: Reproduced with permission from [127]. Copyright 2024, Wiley-VCH. Hybrid: Reproduced with permission from [132]. Copyright 2020, Wiley-VCH. Microstructure design: Reproduced with permission from [75]. Copyright 2022, Wiley-VCH. Environmental stability design: Reproduced with permission from [181]. Copyright 2021, Royal Society of Chemistry. Mechanical property design: Reproduced with permission from [185]. Copyright 2024, Elsevier. Self-healing design: Reproduced with permission from [196]. Copyright 2023, Springer. Wearable electronics: Reproduced with permission from [74]. Copyright 2023, Wiley-VCH. HMI: Reproduced with permission from [203]. Copyright 2022, Elsevier. Environmental monitoring: Reproduced with permission from [43]. Copyright 2024, Royal Society of Chemistry.

In the last few years, several review articles have been published on hydrogel sensors or ionic self-powered sensors, which also contain review articles on hydrogel self-powered sensors [48–52]. For example, Li et al. [49] reviewed recent advances in hydrogel self-powered sensors in terms of both self-powered mechanisms and applications of the sensors, where hydrogels include electronic conductive hydrogels and ionic conductive hydrogels. Additionally, Zhao et al. [51] introduced the development of ionic hydrogels for self-powered tactile sensors in terms of both the basic properties of ionic hydrogels and the self-powered mechanism. However, the development of self-powered sensors utilizing ionic hydrogels based on ion transport mechanism has rarely been more fully and systematically reviewed. This review focuses on the latest research progress of ionic hydrogels in self-powered sensors and provides a more detailed and comprehensive categorization and summary focusing on the 3 aspects of self-powered mechanisms, structural configurations, and performance design of the sensors, which is more targeted and systematic (Fig. 1). In the “Self-Powered Mechanisms” section, a detailed introduction to the energy conversion mechanisms available for ionic hydrogel self-powered sensing systems is provided, including piezoelectric, triboelectric, ionic diode, moist-electric, thermoelectric, potentiometric transduction, and hybrid modes. Next, in the “Structural Engineering” section, we focus on the structural engineering of IHSS, especially the configuration and performance design of the ionic hydrogel sensing layer, including microstructure design, environmental stability, mechanical properties, and self-healing design. Subsequently, the “Applications” section presents new applications based on IHSS, such as wearable electronics, human–machine interaction (HMI), environment monitoring, and medical diagnostics. Lastly, we discuss the existing limitations of IHSS and look forward to its potential development trends.

## Self-Powered Mechanisms

Ionic hydrogels have been widely regarded as the preferred material in fabricating flexible sensors on account of their mechanical performance and physical properties that match biological tissues, as well as their customizable conductivity [53]. However, most existing hydrogel sensors require additional power sources, greatly limiting the potential application fields. In order to break through this limitation, the self-powered sensing principle has become a key exploration direction to solve the problem of autonomous operation of sensors. With the innovation of energy conversion technology, the development prospects of IHSSs with sustainable energy supply are becoming increasingly clear. Unlike other sensors, IHSS converts various external stimuli (energy inputs) into electrical signals (energy outputs) via ion movement, which generates charge separation and potential difference. Over the years, various kinds of IHSS have been derived. In this section, different self-powering mechanisms of IHSS, including piezoelectric, triboelectric, ionic diode, moist-electric, thermoelectric, potentiometric transduction, and hybrid modes, are discussed.

### Piezoelectric effect mechanism

**Fig. 2.**
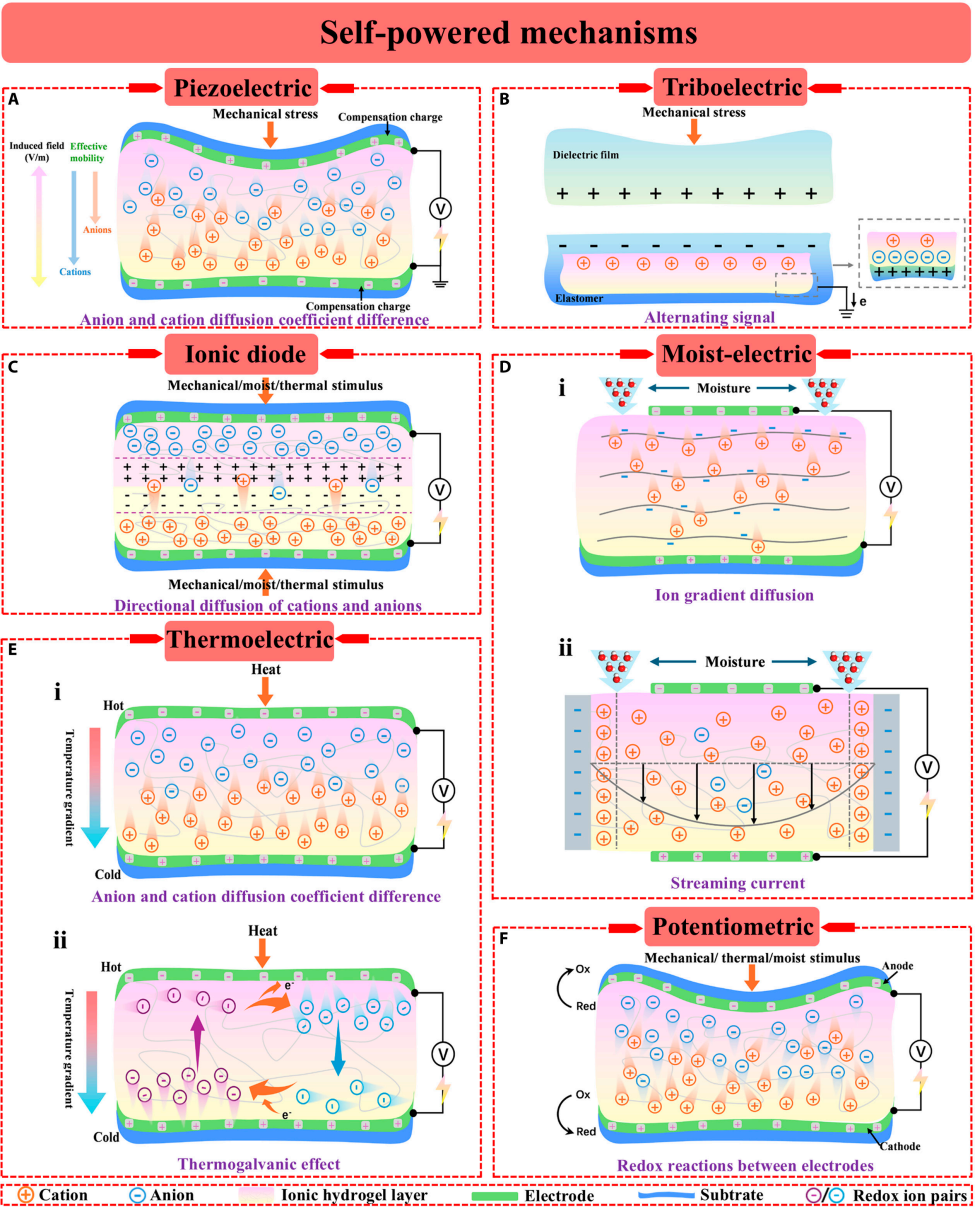
Self-powered mechanisms of IHSS. (A) Piezoelectric. (B) Triboelectric. (C) Ionic diode. (D) Moist-electric. (E) Thermoelectric. (F) Potentiometric.

Ionic hydrogels are materials consisting of the polymer network structures and are rich in water molecules and ions, making it suitable as a functional layer material for pressure sensors. The conventional piezoelectric phenomenon is defined as that when the piezoelectric substance is subjected to applied stress, its internal dipole pairs split, and the induced polarization phenomenon forms complementary positive and negative charges on the material interface, thereby inducing a potential difference [54–56]. Unlike the electronic conduction mechanism of conventional conductors, the conduction mechanism of ionic hydrogels is ions. As illustrated in Fig. 2A, anions and cations within the ionic hydrogels exhibit different mobility in response to external pressure, resulting in a net charge imbalance and thus an electrical signal output [57]. This phenomenon is comparable to the piezoelectric phenomenon and is called piezoelectric ion effect [58]. The piezoelectric effect is a newly available signal/energy generation mechanism, providing an attractive solution for the fabrication of self-powered sensors.

**Fig. 3.**
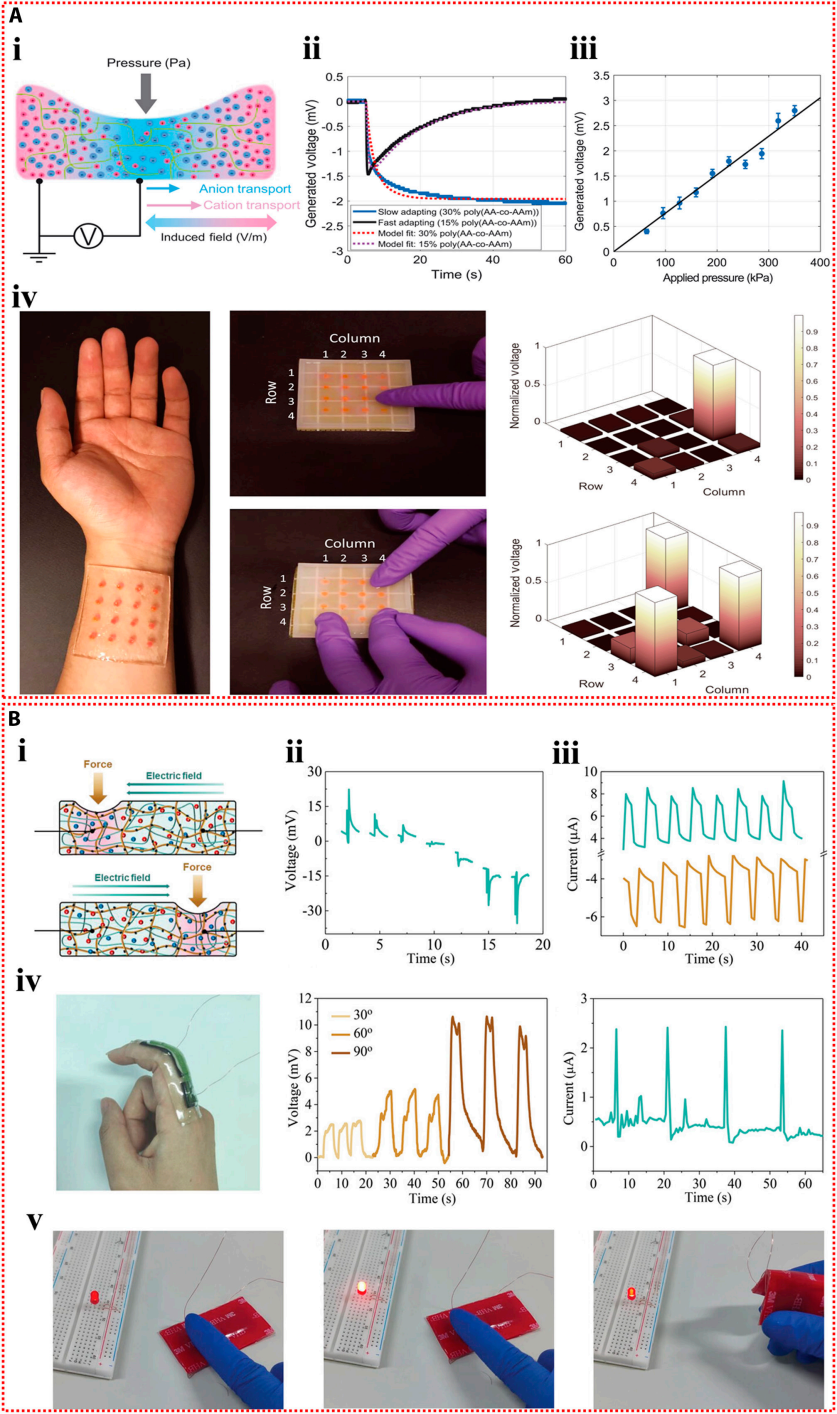
Self-powered mechanisms based on piezoelectric effect. (A) Investigation of piezoelectric properties based on poly(acrylic acid-co-acrylamide) hydrogels. (i) Schematic illustration of a polymer gel undergoing indentation, displaying differential ionic movement and field, where the smaller red cations are moved through the green polymer chain network more rapidly than the blue anions, resulting in an imbalance of charges and the creation of an electric field. (ii) Voltage response of poly(acrylic acid-co-acrylamide) hydrogels during step compression at 20 kPa. (iii) Relationship between the peak voltage produced and the applied pressure. (iv) Photograph and generated voltage histograms of a 16-element piezoelectric mechanoreceptor array mounted on the wrist that detects single and multiple touches. Reproduced with permission from [58]. Copyright 2022, Science. (B) Study of piezoelectric performance based on SnSe nanosheets–dual-network hydrogels. (i) Illustration of force-induced ion aggregation and electric field generation in the hydrogel. (ii) The resulting output voltage profiles for strain moving from the positive to the negative electrodes. (iii) Piezoelectric current curves under strain near 2 electrodes. (iv) Picture of the hydrogel strain sensor used for finger bending monitoring and the resulting output voltage and current signals. (v) Photographs of lighted LED powered by the piezoelectric hydrogel under low pressure, high pressure, and bending. Reproduced with permission from [59]. Copyright 2023, Wiley-VCH.

Dobashi et al. [58] carried out a comprehensive investigation of the piezoelectric characteristics of ionic hydrogels and demonstrated that when hydrogel materials were subjected to compression, an ionic gradient was produced as a result of asymmetry in migration rate of anions and cations, thereby generating a pressure signal. An indentation experiment was designed to explore the microscopic mechanisms of piezoelectric phenomenon and sensing application (Fig. 3Ai). During compression, smaller cations migrated faster than anions, causing a charge imbalance and creating an electric field. Reducing the hydrogel polymer content could dramatically accelerate the responsive behavior (Fig. 3Aii). The peak voltage generated increased with the applied pressure (Fig. 3Aiii). The fabricated artificial mechanoreceptor constituted a poly(acrylic acid-co-acrylamide) hydrogel semisphere, which was circumscribed with polyacrylamide hydrogel swollen in 0.1 M KCl on a plane, forming a 4 × 4 touch sensor array. Single-point and multi-point touches (approximately 100 g of force) on the sensor array by a finger produced a voltage variation of around −10 mV, which was superimposed on the basic Donnan potential of −50 mV (Fig. 3Aiv).

Li et al. [59] fabricated a structurally optimized ionic hydrogel material containing SnSe nanosheets with a piezoelectric coefficient as high as 1,780 nV Pa^−1^ and −7.21 nA Pa^−1^. The piezoelectric phenomenon within hydrogels originates from the unbalanced charge associated with strain-induced ion flow. The diffusion speed of large-sized anions is comparatively slower, causing them to accumulate in the stressed area, further developing an electric field pointing in the direction of stress (Fig. 3Bi). When the stress location moved through positive to negative, the forward voltage progressively decreased to approximately 0 mV, following a shift to negative voltage, and continued to increase (Fig. 3Bii). The stability and repeatability of the piezoelectric signal were crucial for sensing applications. Multi-cycle strain tests near the electrodes on the SnSe–hydrogel showed repeatable output current (Fig. 3Biii). This ionic hydrogel could be employed as a sensor to identify flexion movement of fingers, capable of distinguishing the joint movements (Fig. 3Biv). Moreover, the hydrogel device was connected to a light-emitting diode (LED) via a voltage amplifier. The brightness of the LED can be tuned by pressure-induced piezoionic voltage. The LED is less bright under low pressure and more bright under high pressure. The LED can also be lighted by bending the hydrogel strain sensor (Fig. 3Bv). Moreover, Odent et al. [60] achieved the preparation of stacked ionic components through 3D printing and proposed a novel self-powered ionic electronic sensor utilizing tactile-triggered ion charge separation mechanism. Polyelectrolyte hydrogels rich in free K^+^ or Cl^−^ were prepared, and in-depth exploration of the piezoelectric properties of these hydrogels was conducted. In the designed dual-compartment hydrogel system, the ionic concentrations in each compartment were distinct, which substantially established the ionic concentration difference. At the 50% compression deformation setting, an output signal of 70 mV can be observed. The piezoelectric effect originated from the output voltage produced by ion separation with different migration rates under mechanical stimulation. The piezoelectric behavior was closely related to the type of ions and charge density. The assembled tactile sensor was mounted to a finger to achieve the touch feedback function and serve the identification task.

The dynamic behavior of ions observed in piezoelectric ionic hydrogel systems is comparable to the signal production and delivery mechanisms attached to living organisms, which gives this material great potential in the development of bionic self-powered sensors. The piezoelectric mechanism has gradually emerged as a simple and effective method of harvesting surrounding mechanical energy owing to the merits of low cost, simple fabrication process, and the ability to be integrated with other sensing technologies. Moreover, ionic hydrogel-based piezoelectric sensors allow for reversible and repetitive deformation, which is expected for long-term utilization. Due to the heavy reliance of piezoionic effects on ion mobility and the slow rate of ion transfer, the piezoionic sensors exhibit relatively long response and recovery time. Furthermore, the electrical output of piezoionic sensors is generally low, making it crucial in improving piezoionic materials’ output performance. Furthermore, the precision control and environmental adaptability of the instrumentation are essential to guarantee measurement reliability and withstand the disruption of external environmental factors. Although the piezoelectric output signals in response to mechanical deformations are limited by the intrinsic properties of the piezoelectric material, ionic hydrogel-based piezoelectric sensors may compensate for a part of the specific applications that require high sensitivity, compliance, adaptation, spatiotemporal interaction, etc.

### Triboelectric mechanism

Triboelectric sensor is another typical self-powered sensor based on the sensing of external mechanical stimuli. Unlike self-powered sensors based on piezoelectric mechanism, sensors based on the triboelectric mechanism can generate the larger voltage response. Triboelectric nanogenerator (TENG), as an emerging energy harvesting mechanism, relies on contact electrification and electrostatic induction to respond to mechanical stimulation and generate corresponding electrical signals, and has been extensively explored to achieve energy harvesting and self-powered sensing [61–63]. Therefore, sensing can be performed without an external power supply. TENG could be categorized into multiple working modes according to the electrode arrangement and movement type, consisting of independent triboelectric layer, lateral sliding, vertical contact separation, and single electrode [64]. Charge transfer takes place between the surfaces of 2 materials when their electron affinities are different and they come into touch with each other and separate (Fig. 2B). The charges or electrons flow through an external circuit, usually producing an alternating current output, to maintain electrostatic equilibrium [65,66]. The continuous alternating current power output can be realized by continuously repeating the contact and separation cycles [67,68]. By replacing the electrode (or friction layer) layer with ionic material, ionic TENG can be easily obtained. Ionic hydrogels with shape adaptation, transparency, high stretchability, and adjustable conductivity are undoubtedly a good candidate for flexible electrodes in TENG and its sensing applications [69,70]. Ionic hydrogels exhibit fast internal ion transfer and separation by the incorporation of ions as charge carriers, resulting in improved triboelectric charging performance [61,71].

**Fig. 4.**
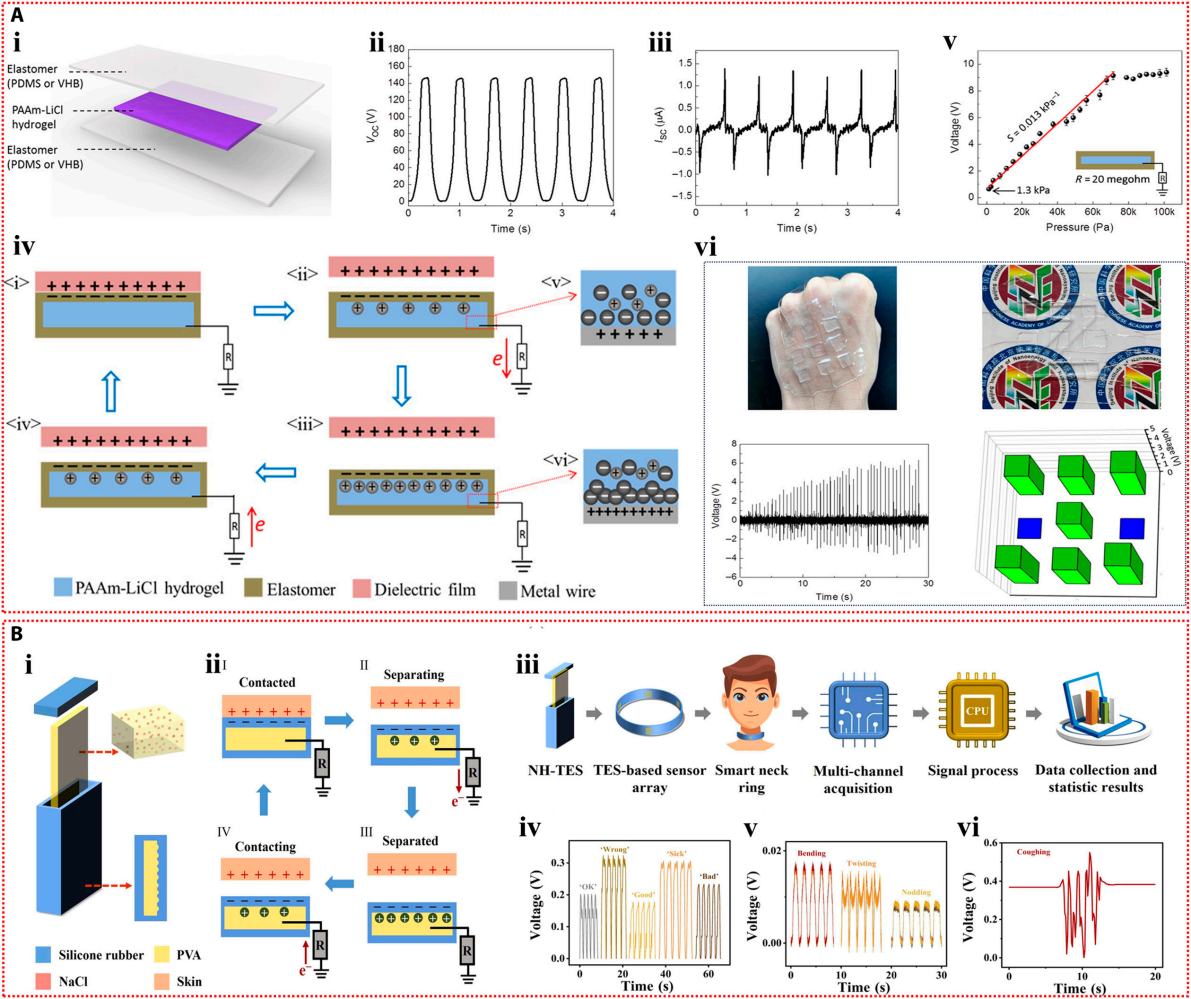
Self-powered mechanisms based on triboelectric effect. (A) Study of triboelectric performance based on the polyacrylamide hydrogel containing lithium chloride. (i) Schematic diagram of the device with sandwich structure. The generated output voltage (ii) and current (iii) of the device. (iv) Schematic representation of the working principle of the device. (v) Variation of the peak voltage across the connected resistor with external pressure. (vi) The voltage signals generated by pressing the TENG tactile sensor with 9 pixels affixed to the curved hand and the z-shaped acrylic sheet. Reproduced with permission from [72]. Copyright 2017, Science. (B) Investigation of triboelectric properties based on the sodium chloride/poly(vinyl alcohol) ionic hydrogel. The structure (i) and operating mechanism (ii) of the TENG device. (iii) The detection process of the hydrogel TENG smart neck ring for monitoring neck motions. The output voltage signals of the smart neck ring during talking (iv), rotating (v) and coughing (vi). Reproduced with permission from [73]. Copyright 2023, Elsevier.

**Fig. 5.**
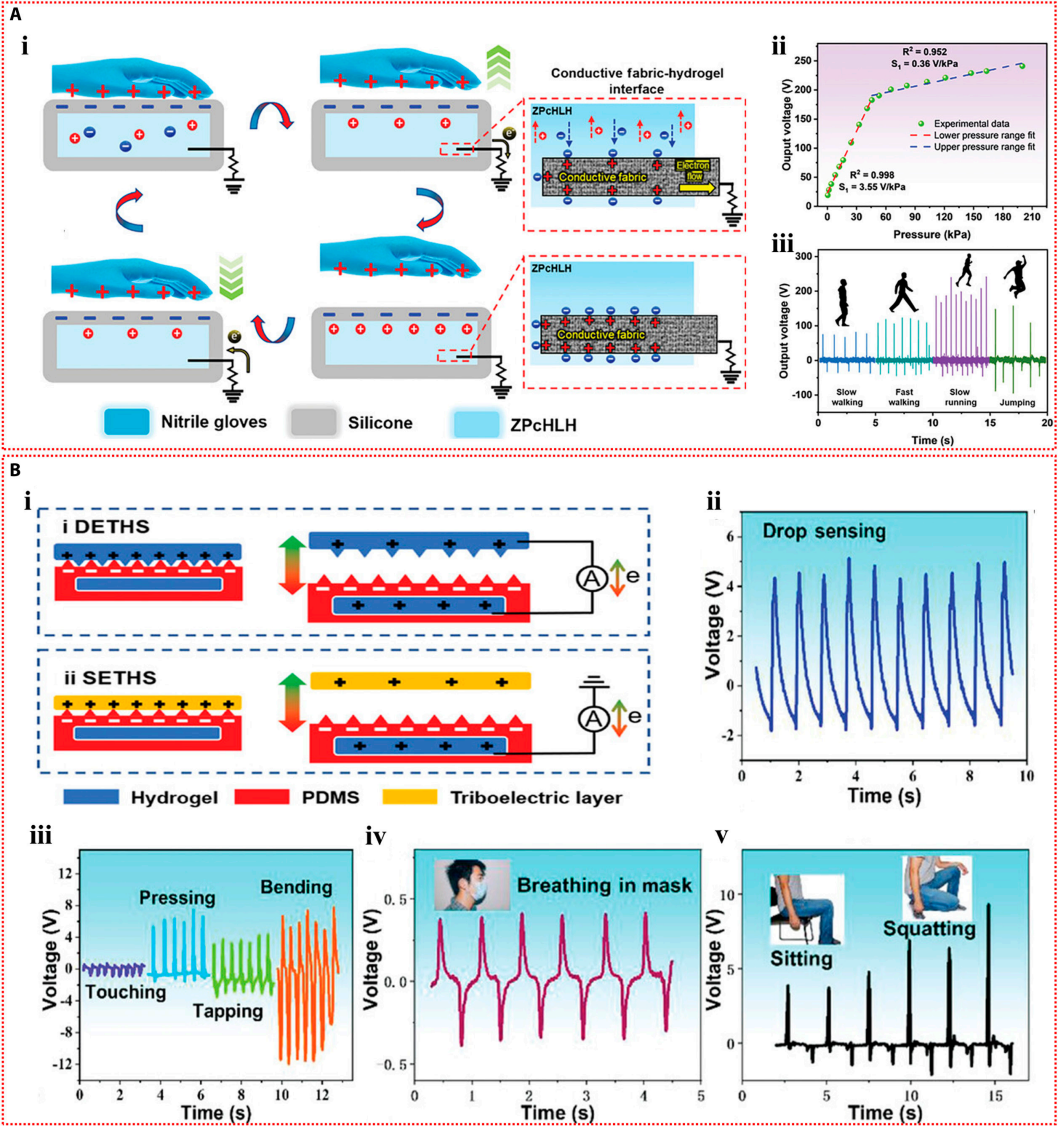
Self-powered mechanisms based on triboelectric effect. (A) Investigation of triboelectric performance based on the poly(acrylamide)-co-hydroxyethyl acrylate composite hydrogel containing lithium chloride. (i) Schematic of the working principle of the TENG device operating in the single-electrode mode. (ii) The output voltage of the TENG-based self-powered sensor versus external pressure. (iii) The response signals of the sensor in the slow walking, fast walking, slow running, and jumping states of the human body. Reproduced with permission from [74]. Copyright 2023, Wiley-VCH. (B) Study of triboelectric properties based on the polyacrylamide/carrageenan hydrogel containing lithium bromide. (i) Schematic diagram of the operating mechanism of the double-electrode triboelectric sensor and the single-electrode triboelectric sensor. (ii) Response signals of the single-electrode triboelectric sensor in water droplet sensing. (iii) Response signals of the 2-electrode triboelectric sensor during touching, pressing, tapping, and bending. Application of the 2-electrode triboelectric sensor for monitoring human respiration (iv) and knee motion (v). Reproduced with permission from [75]. Copyright 2022, Wiley-VCH.

The first ionic hydrogel-based TENG was presented by Pu et al. [72] in 2017 and had excellent stretchability and transparency. The design made use of an elastomer film as the electrification layer and a polyacrylamide hydrogel with lithium chloride as the flexible electrode (Fig. 4Ai). The peak output of 145 V and 1.5 μA was attained by this flexible ionic hydrogel-based TENG (Fig. 4Aii and iii). The device operated in single-electrode mode because the ionic hydrogel was grounded via a metal wire that was attached to an external load (Fig. 4Aiv). The TENG showed a minimum detection limit of roughly 1.3 kPa and a sensitivity of 0.013 kPa^−1^ when used as a pressure sensor (Fig. 4Av). The soft and transparent ionic hydrogel TENG sensors can be applied as touch/pressure sensing artificial electronic skin (Fig. 4Avi). Since then, the TENG sensors based on ionic hydrogels have gained more attention and experienced rapid development and applications. Luo et al. [73] developed a wearable TENG sensor using a sodium chloride/poly(vinyl alcohol) ionic hydrogel to monitor driver conditions. The hydrogel was used as the electrode in the pocket structure of the sensor, while silicone rubber was used as the substance that caused negative friction. Furthermore, a porous silicone rubber film was produced, which resulted in a curved contact surface between the friction layer and the hydrogel electrode (Fig. 4Bi). Due to the difference in electronegativity, repeated contact and separation between human skin and the silicone rubber film produced repetitive alternating electrical signals, and the device operated in the single-electrode mode (Fig. 4Bii). Four self-powered sensors were integrated into a strip-like sensor array as a smart neck ring to monitor the movement conditions of the neck. The 4 sensors were distributed at different locations of the neck (Fig. 4Biii). After being transformed into digital signals by the neck ring, the analog electrical impulses were sent to a computer for data processing. The data were then processed and transferred to a terminal device to record the motions of the neck for further examination. The output voltage profiles corresponding to different neck motions, including speaking, rotating, and coughing, are shown in Fig. 4Biv to vi. The substantial potential of self-powered sensors in smart transportation applications is highlighted by this study. Furthermore, Rahman et al. [74] fabricated a flexible and durable TENG electrode by incorporating the nanofiller zeolitic imidazolate framework-8 (ZIF-8) and lithium chloride electrolyte into the poly(acrylamide)-co-hydroxyethyl acrylate hydrogel matrix. Through the combined effects of contact charging and electrostatic induction between 2 triboelectric layers, the device generates electricity in single-electrode mode. In addition to facilitating ion transport, the ionic hydrogel electrode electrostatically shields the friction charges produced on the negative friction material (Fig. 5Ai). The maximum output of the hydrogel-based TENG was 232 V and 56.3 mA m^−2^. With its high sensitivity and wide dynamic range, this TENG can be used as an independent pressure sensor for wearable technology (Fig. 5Aii). Furthermore, by examining changes in voltage and frequency, the self-powered sensor allowed for the tracking and identification of human motion when it was inserted into the insole (Fig. 5Aiii).

Ionic hydrogel can also be used in TENG as a triboelectric layer in addition to a single electrode. Tao et al. [75] developed an ionic hydrogel by incorporating lithium bromide into a dual-network hydrogel made of polyacrylamide and carrageenan. This triboelectric layer and electrode were made of this microconical ionic hydrogel. The top ionic hydrogel layer of the double-electrode TENG separated from the bottom PDMS friction layer when repeatedly subjected to external force (Fig. 5Bi), producing electrical signals with a maximum output voltage of 18 V through triboelectrification and electrostatic induction effects. The TENG could also operate in single-electrode mode. This flexible TENG, which has a high sensitivity of up to 45.97 mV Pa^−1^, was created as an autonomous tactile sensor for wearable applications. The self-powered sensor’s ability to recognize subtle stimuli, like continuously flowing water droplets on the device’s surface, is demonstrated in Fig. 5Bii. Additionally, it successfully tracked volunteers’ knee joint movements, breathing, and a variety of hand movements (Fig. 5Biii to v).

With unique benefits such as low cost, wide material availability, easy fabrication, and diverse designability, TENG is another effective component as an environmentally friendly and sustainable power source for a variety of sensors. Researchers have made great strides in the creation of flexible and stretchable TENG in the last few years [76–79]. The use of ionic hydrogels has facilitated the creation of stretchable and transparent electrodes, playing a crucial role in advancing TENG self-powered sensors. Moreover, considering the high biocompatibility, conformability, and self-healing of ionic hydrogel materials, ionic TENG sensors have potential to be easily attached to human skin or even implanted inside the human body. However, ionic hydrogel-based TENG also faces many challenges. For instance, TENG typically exhibits relatively low output power density at low frequencies. The charge conversion efficiency generated by the friction of hydrogel-based TENG still requires improvement. Additionally, further optimization of the materials’ structure and properties is necessary to enhance the energy conversion efficiency. Moreover, hydrogels may suffer from material fatigue, attrition, and failure during prolonged operation. Besides, the sensitivity to ambient humidity is one of the problems that need to be overcome. Therefore, more research and improvements will be necessary to achieve widespread application and commercialization.

### Ionic diode-based mechanism

Recently, ionic diode devices consisting of polycationic and polyanionic ionoelastomers have demonstrated great promise as self-powered sensors [80,81]. Similar to self-powered sensors based on piezoelectric mechanism, ion diode-based self-powered sensors utilize the slow diffusion of mobile ions within the material in response to mechanical stimuli, which allows the sensor to operate at low frequencies. Nevertheless, in contrast to the single pressure stimulus in the piezoelectric mechanism, ion diode-based self-powered sensors can respond to a variety of external stimuli, such as mechanical stress, humidity, and temperature. When diffusion equilibrium is reached, hydrogel ionic diodes form an initial built-in potential by using the directional diffusion of cations and anions to create a depletion region akin to a p-n semiconductor junction [82]. As displayed in Fig. 2C, the inherent voltage across the depletion region is intensified by the diffusion of ions caused by external stimuli. This process effectively converts different types of energy into electrical energy.

**Fig. 6.**
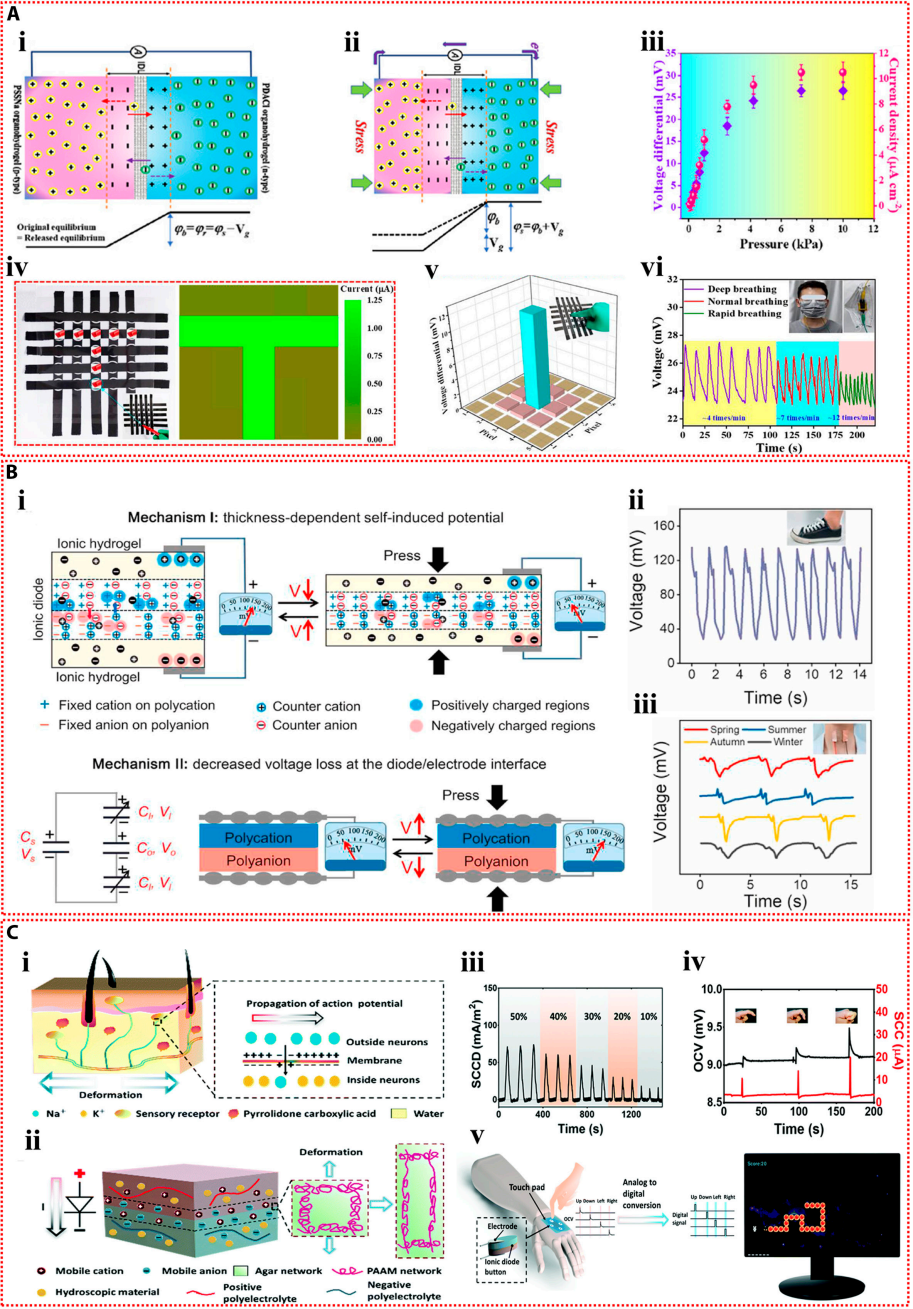
Self-powered mechanisms based on ionic diode. (A) Study of ionic diode based on the agarose hydrogels containing polyelectrolytes, MXene, and ethylene glycol. (i) Schematic diagram of the mechanism of the hydrogel ionic diode in the initial state (i) and stressed state (ii). (iii) Output voltage and current produced at various pressures. (iv) Painting the letter “T” on the ionic diode self-powered sensor array with the tip of a pen and the resulting pressure imaging. (v) Diagram of the voltage signal mapping produced by pressing the center pixel of the sensor array. (vi) Response signals of the hydrogel ionic diode to different frequencies of human breathing. Reproduced with permission from [83]. Copyright 2023, Wiley-VCH. (B) Investigation of ionic diode based on the polyacrylamide hydrogels containing sodium methacrylate alginate and chitosan methacrylate. (i) The operating principle of the self-powered hydrogel ionic diode device. Response signals generated by the ionic diode as a self-powered skin monitoring the walking motion (ii) and the vibration of the vocal cords as the volunteer speaks (iii). Reproduced with permission from [84]. Copyright 2022, Elsevier. (C) Study of ionic diode based on the agarose–polyacrylamide double-network hydrogels containing polyelectrolytes and ethylene glycol. (i) Illustration of directional transport of ionic signals within sensory neurons in human skin. (ii) Schematic diagram of the ionic diode self-powered skin. (iii) Output current responses under repetitive compression at various strain levels. (iv) Voltage and current response signals of the ionic diode self-powered sensor for finger joint bending monitoring. (v) Schematic of a touchpad consisting of self-powered ionic diode buttons attached to a human hand to control the game of greedy snake. Reproduced with permission from [85]. Copyright 2019, Royal Society of Chemistry.

Yin et al. [83] prepared 2 types of polyelectrolyte hydrogels by introducing sodium polystyrene sulfonate and poly(diallyldimethylammonium chloride) into hydrogels containing agarose, MXene, and ethylene glycol, respectively, and then assembled them to form the ionic diode device. The 2 polyelectrolytes in the hydrogel dissociate sodium and chloride ions, respectively. These mobile ions diffuse across the bilayer hydrogel interface, creating a depletion zone and an initial built-in potential. This intrinsic potential is enhanced in response to external pressure or humidity, which is the basis for the ionic diode’s power generation mechanism (Fig. 6Ai and ii). The output of the ionic diode self-powered sensor increased consistently with rising pressure across a broad pressure range, achieving a high current output of up to 10.10 μA cm^−2^ (Fig. 6Aiii). Hydrogel ionic diodes showed promise for tactile sensing and self-powered humidity sensor applications. The self-powered sensor can not only detect the slide of a pen tip and the touch of a finger but also monitor the respiratory status in real time based on the moisture released by human breath (Fig. 6Aiv to vi). Du et al. [84] fabricated a polyanionic hydrogel and a polycationic hydrogel by introducing sodium methacrylate alginate and chitosan methacrylate into the polyacrylamide hydrogel, respectively. The ionic diode device was then assembled by employing the 2 stretchable ionic hydrogels as electrodes. Concentration gradient-driven diffusion of the mobile counterions results in the generation of built-in potential. The device functions through a thickness-dependent self-induced potential and potential loss at the interface between the electrode and ionic diode (Fig. 6Bi). With the help of this ionic diode gadget, which can sense pressures and strains from the environment on its own and respond to them, heel compression and faint vocal cord vibrations can be precisely monitored (Fig. 6Bii and iii).

Motivated by the operational principles of human skin sensory neurons, Ying et al. [85] developed a stretchable and environmentally stable ionic diode skin (Fig. 6Ci and ii). Bilayer ionic hydrogels were prepared by introducing positively and negatively charged polyelectrolytes, as well as hygroscopic ethylene glycol, into the double-network hydrogel composed of agarose and polyacrylamide. To achieve electromechanical conversion, the ionic diode translates its deformation into variations in intrinsic potential and the diffusion of free ions. The ionic diode device’s output current increases as strain does (Fig. 6Ciii). This ionic diode skin can be used as a wearable, self-powered strain sensor (Fig. 6Civ), especially for tracking movements of finger joints. In addition, the touchpad consisting of self-powered ionic diode buttons can control a game, showing its potential for human–computer interaction applications (Fig. 6Cv).

Hydrogel ionic diodes can provide longer current duration and higher current density when subjected to mechanical stimulation at low frequencies. Nevertheless, the output value of these devices is relatively small. Improving the rectification ratio of the ionic diode and reaching higher output levels will be a major challenge in the future. Furthermore, the cumbersome preparation of bilayer ionic hydrogels and the unfavorable delamination between diode components are also limitations of the ionic diode.

### Moist-electric mechanism

The response signals of IHSS based on piezoelectric, triboelectric, and ion diode-based mechanisms are mainly derived from the conversion of mechanical energy to electrical energy. Apart from responding to common mechanical stimuli of pressure or tensile force, IHSS can also sense other external environmental stimuli. With its abundant presence in the atmosphere, moisture holds great potential as a promising source of clean, green, and renewable energy [86,87]. The chemical potential of gaseous water can be converted into electricity using the developing technology known as moist-electric generation (MEG), providing a free power source that may power the rising wave of wearable electronics [88–90]. Furthermore, moisture-sensitive MEG devices can function as autonomous humidity sensors, which is helpful for monitoring human respiration and noncontact human–computer interaction [91,92]. Because of their excellent hygroscopic properties, low cost, and ease of preparation, ionic hydrogels have been developed for MEG applications [93,94]. Ionic hydrogel can be dissociated to generate free-moving ions after absorbing moisture, and the directional migration of the free-moving ions establishes the potential difference, thus realizing the moist-electric conversion [95,96]. As shown in Fig. 2D, ion transport can be categorized into 2 primary types: the movement of counter ions in confined water within the electric-double layer (EDL) and the migration of dissociated mobile ions from functional groups, which is motivated by the concentration gradient [97,98].

**Fig. 7.**
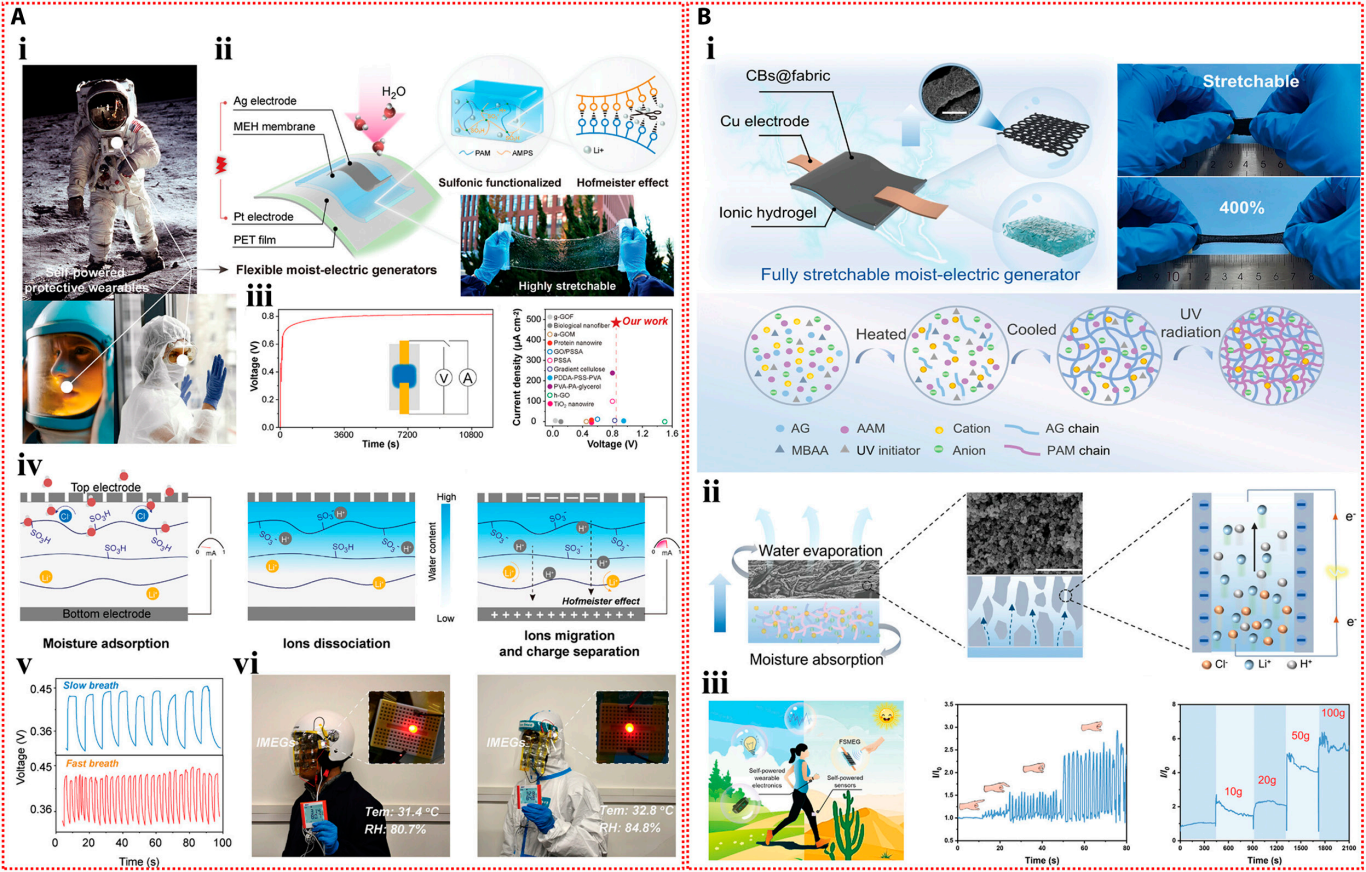
Self-powered mechanisms based on moist-electric. (A) Investigation of moist-electric performance based on the poly(acrylamide-co-2-acrylamide-2-methyl propane sulfonic acid) hydrogel containing lithium chloride. (i) Potential applications of MEG in the self-powered protective wearables. (ii) Illustration of the device structure of the MEG and an image of the highly stretchable ionic hydrogel membrane. (iii) The curves of output voltage and current of the device as a function of time under various relative humidity levels. (iv) Illustration of the power generation mechanism of the device. (v) Response signals for monitoring respiration at different frequencies by the MEG as a self-powered sensor. (vi) The devices integrated into closed helmet and medical suit by converting the moisture energy from respiration into electricity to light up a red LED. Reproduced with permission from [39]. Copyright 2023, Wiley-VCH. (B) Study of moist-electric properties based on the agarose–polyacrylamide double-network hydrogel containing lithium chloride. (i) Schematic of the structure and composition of the device and the images of its stretchability. (ii) Schematic representation of the operating principle of the device. (iii) Schematic diagram of the device powering self-powered wearable electronic devices and acting as the self-powered sensor, and the response of the sensor at different bending angles of the finger and weight. Reproduced with permission from [99]. Copyright 2023, Wiley-VCH.

Self-sustaining wearable technology can be powered by moist-electric devices that capture the moisture energy produced by breathing humans (Fig. 7Ai). Using acrylamide, 2-acrylamide-2-methyl propane sulfonic acid, and LiCl as raw materials, Zhang et al. [39] created a stretchable and flexible moist-electric generator (Fig. 7Aii). At 80% relative humidity, the device’s maximum electrical output was 0.81 V and 480 μA cm^−2^ (Fig. 7Aiii). The mechanism of power generation is as follows: Under an asymmetric humidity gradient, the concentration gradient causes free hydrogen ions that have dissociated from sulfonic acid groups on the hydrogel polymer chains to migrate directionally, resulting in a marked charge separation. Lithium ions also cause the Hofmeister effect to break hydrogen bonds between polymer chains, which widens ion transport channels and increases electrical output (Fig. 7Aiv). This device has the potential to be used in human health monitoring since it can monitor respiratory frequencies as a self-powered sensor (Fig. 7Av). Moreover, it can transform breathing moisture energy into electricity when integrated into a wearable device, which is enough to turn on a red LED (Fig. 7Avi).

By combining a hygroscopic ionic hydrogel containing lithium chloride with cotton knit fabric coated in carbon black and employing symmetrical copper (Cu) electrodes, Wen et al. [99] created a fully stretchable moist-electric generator (Fig. 7Bi). The apparatus demonstrated a 400% strain capacity and consistently generated an electrical output of about 50 μA and 0.3 V. When the device is exposed to the environment, the ionic hydrogel hygroscopic layer inside can quickly absorb water and separate anions and cations. The cations will be drawn to the dissociated ions and form an EDL at the liquid–solid interface as they flow directionally with the water through the negatively charged nanochannels in the upper evaporation layer. The EDL will be moving in response to the evaporation force, producing an electrical output (Fig. 7Bii). Furthermore, when the device is under pressure, the hygroscopic and evaporative layers get thinner. This shortens the counterion diffusion distance, which raises the output current. As such, the apparatus can be employed as an autonomous sensor to track the flexion of fingers and to distinguish between various weights (Fig. 7Biii).

Ion hydrogels based on MEG show great promise for power supply and self-powered electronic devices. Because of their quick reaction to temperature, relative humidity, and moisture flow rate changes, one of their most prominent uses is in self-powered sensors for monitoring environmental parameters, such as skin moisture and respiration rate [100]. These sensors have the ability to directly capture atmospheric energy, which increases their potential for portability and miniaturization by allowing them to operate without the need for an external power source. Although MEG-based sensors demonstrate potential across various applications, several challenges remain before they can be commercially viable. These challenges include improving response rate, sensitivity, device packaging, and data transmission. The output current signal of the sensor is notably more sensitive to ambient humidity than the output voltage. Monitoring changes in the current signal can further improve the response speed. Additionally, achieving a stable humidity gradient within the device and realizing continuous power supply applicable across all regions are challenges in the field of ionic hydrogel moisture-electric generation. Further efforts are needed to overcome both challenges.

### Thermoelectric mechanism

In addition to the moisture, renewable heat from the surroundings or the human body can also be converted into electricity by the IHSS, providing a rational solution for the self-driving of the sensors. Utilizing low-grade thermal energy to provide effective energy supply has become a promising pathway to achieving sustainable energy development [101–103]. In traditional electronic thermoelectric materials, the thermoelectric potential is generated by the migration of electrons under a temperature gradient [104]. In contrast, in ionic thermoelectric materials, the potential is created by the migration of ions under a temperature gradient, a phenomenon known as the ionic thermodiffusion effect, or the Soret effect [105,106]. As presented in Fig. 2E, besides this method of generating thermoelectric potential, ionic thermoelectric materials have another source: When temperature-controllable redox ion pairs are introduced into the material, redox reactions occur at the electrodes, converting thermal energy into electrical energy through changes in entropy during the reactions. This effect is known as the thermogalvanic effect [107,108]. Hydrogel electrolyte materials, known for their high ionic conductivity and flexibility, are capable of lowering the overall thermal conductivity of batteries and preventing electrolyte leakage. They therefore hold great promise for producing electrical energy from low-grade thermal energy [109,110]. The temperature differential between the human body and the environment can be used to power thermoelectric generators based on ionic hydrogels, which produce electrical energy from body heat. These gadgets can also function as independent sensors [111,112].

**Fig. 8.**
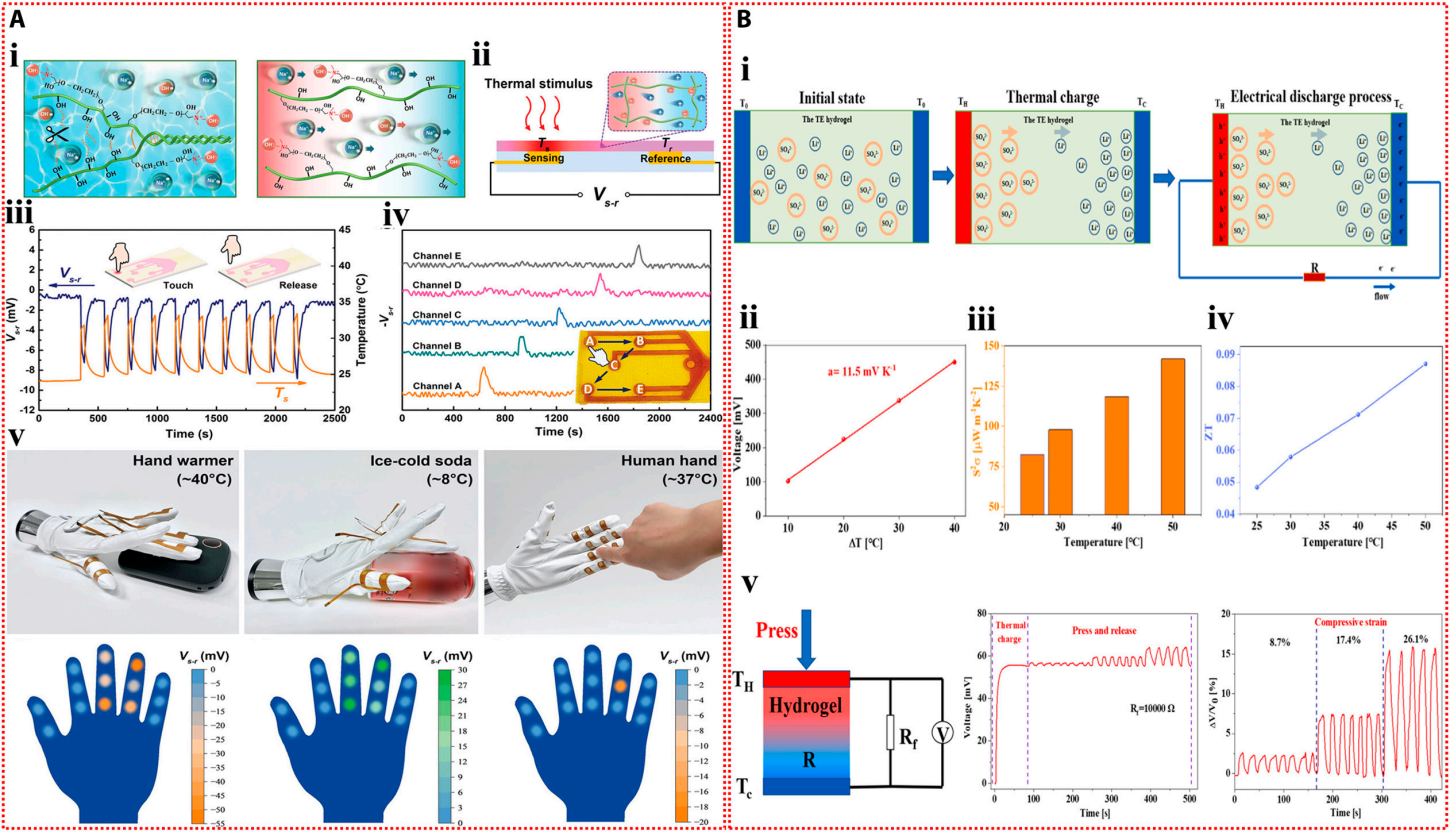
Self-powered mechanisms based on thermodiffusion effect. (A) Study of thermoelectric performance based on the ionic hydrogel containing polyquaternium-10 and sodium hydroxide. (i) Schematic diagram of the power generation mechanism of the device. (ii) Illustration of thermoelectric sensing mechanism based on the ionic hydrogel. (iii) Response signals generated by cyclic touching and moving away of a finger on a single channel. (iv) Response signals generated by successive touching and moving away of a finger on multiple channels in sensor arrays. (v) Photos of an intelligent glove fitted with several thermoelectric sensor arrays upon contact with objects with different temperatures and the corresponding voltage signal distributions. Reproduced with permission from [113]. Copyright 2023, Wiley-VCH. (B) Investigation of thermoelectric properties based on the polyacrylamide–calcium alginate double-network hydrogel containing Li_2_SO_4_. (i) Schematic diagram of the power generation mechanism of the thermoelectric device under temperature difference. (ii) The thermal voltage generated by the device as a function of the temperature difference. Power factor (iii) and the figure of merit (iv) of the thermoelectric device at different temperatures. (v) Schematic diagram of the thermoelectric device connected to an external load resistor as a self-powered strain sensor and the response signals to compressive strains at the temperature difference of 40 °C. Reproduced with permission from [114]. Copyright 2021, Elsevier.

In recent years, thermoelectric technologies based on the thermodiffusion effect have been extensively explored. Han et al. [113] synthesized a green, low-cost ionic hydrogel using polyquaternium-10 and sodium hydroxide as raw materials. As shown in Fig. 8Ai, Na^+^ and OH^−^ disrupted the hydrogen bonds among the molecular chains within the hydrogel, exposing additional cationic quaternary ammonium groups. As Na^+^ moved freely in response to the temperature gradient, the positively charged molecular chains attracted OH^−^ through electrostatic interactions, increasing the thermoelectric output. A thermoelectric sensor array was then constructed based on this ionic hydrogel. When thermal stimuli were applied to the sensing nodes, the imbalance diffusion of Na^+^ and OH^−^ under the temperature gradient generated a potential difference within the hydrogel, revealing the thermal conditions of the sensing area (Fig. 8Aii). Figure 8Aiii demonstrates the excellent repeatability of the potential difference generated by cyclic touch-and-release of a finger on the sensor array. By simultaneously monitoring the signals from all channels when a finger touched a specific sensing node, the location and time of contact between the finger and the sensor array could be determined (Fig. 8Aiv). Furthermore, an intelligent glove integrating multiple thermoelectric sensor arrays was constructed. The intelligent glove, worn on an artificial hand, was able to detect the temperature and touch position of objects by producing different voltage responses when it came into contact with them at different temperatures (Fig. 8Av).

**Fig. 9.**
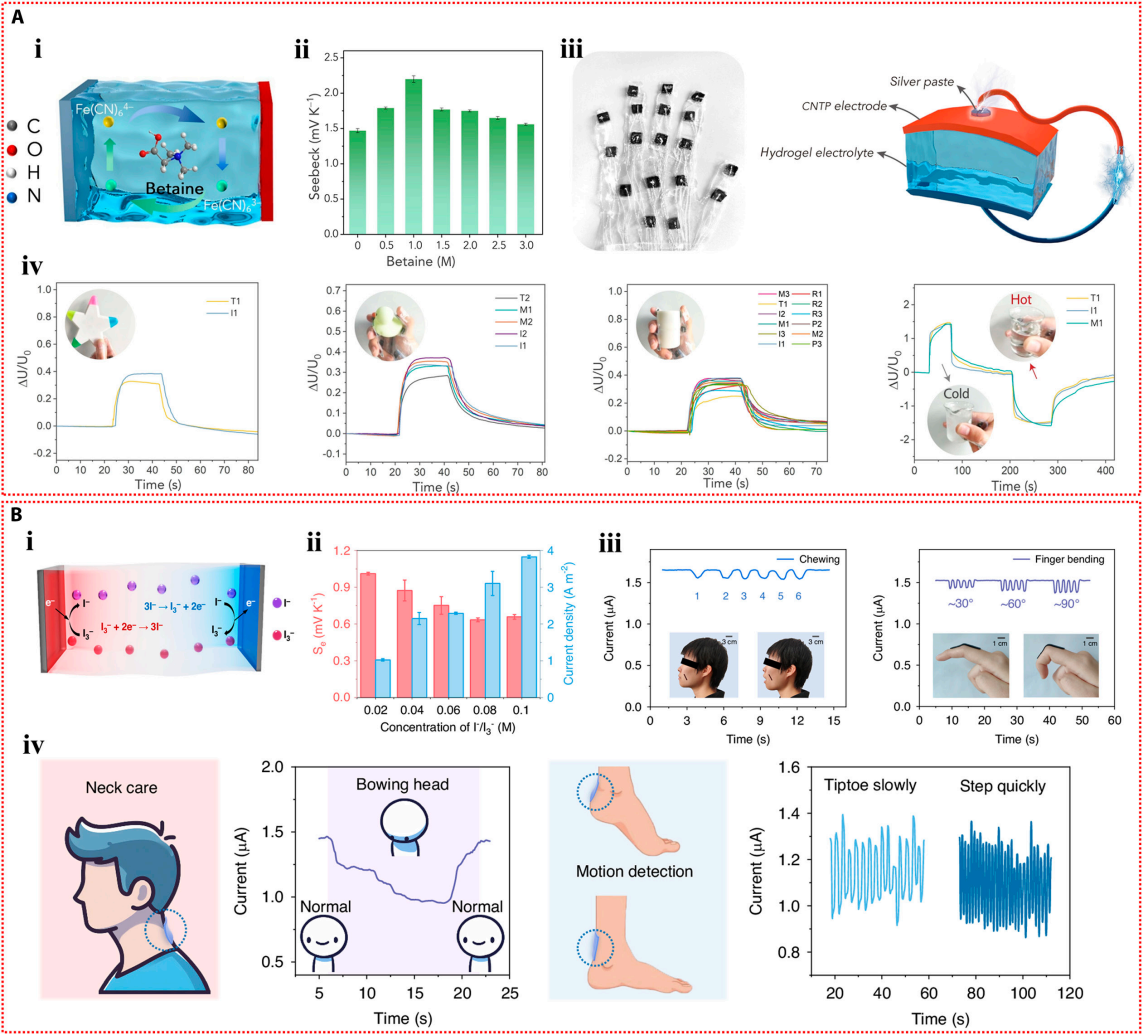
Self-powered mechanisms based on thermogalvanic effect. (A) Investigation of thermoelectric performance based on the gelatin hydrogel containing zwitterionic betaine and Fe(CN)_6_^3−^/Fe(CN)_6_^4−^ redox couple. (i) Illustration of the working mechanism of the thermoelectric device. (ii) The influence of betaine content on Seebeck coefficient. (iii) The photograph of the smart glove composed of 18 thermoelectric devices and the schematic of the structure of a single device. (iv) Voltage signals of the device when the hand is in contact with a pentagram toy, a duck toy, a cylinder, and a hot/cold water cup. Reproduced with permission from [115]. Copyright 2024, Wiley-VCH. (B) Study of thermoelectric properties based on the polyvinyl alcohol hydrogel containing I^−^/I_3_^−^ redox couple. (i) Schematic diagram of the operating principle of the thermoelectric device. (ii) The effect of I^−^/I_3_^−^ content on Seebeck coefficient and current density. (iii) The current responses of the thermoelectric device as the strain sensor in monitoring of chewing and finger bending. (iv) The current responses of the thermoelectric sensor in monitoring the degree of neck flexion and the intensity of foot movement. Reproduced with permission from [116]. Copyright 2024, Springer Nature.

Furthermore, Chen et al. [114] used polyacrylamide, calcium alginate, and Li₂SO₄ to create a conductive hydrogel with superior thermoelectric qualities. As depicted in Fig. 8Bi, due to the thermodiffusion effect, the temperature gradient across the ionic hydrogel caused Li^+^ and SO₄^2−^ ions to migrate from the hot side to the cold side. Since Li^+^ ions are smaller and migrate faster than SO₄^2−^ ions, they accumulated on the cold side, while sulfate ions remained on the hot side, generating a thermoelectric output. The output voltage of the device increased with the temperature difference, achieving a Seebeck coefficient of 11.5 mV K^−1^ (Fig. 8Bii). The power factor of the thermoelectric generator also rose with temperature, reaching a peak of 141.86 μW m^−1^ K^−2^ (Fig. 8Biii). Moreover, the figure of merit for the ionic hydrogel ranged from 0.048 to 0.087 within the temperature range of 25 to 50 °C (Fig. 8Biv). A self-powered strain sensor was constructed by applying the thermoelectric voltage of the ionic hydrogel to drive an external load resistance. The hydrogel’s ability to detect external pressure signals was made possible by the conversion of the relative resistance change that resulted from compressive strain under a temperature gradient into a voltage change across the fixed load resistance (Fig. 8Bv). Moreover, IHSS devices based on the thermogalvanic effect are emerging. Lu et al. [115] prepared a thermoelectric cell based on a gelatin, zwitterionic betaine, and Fe(CN)_6_^3−^/Fe(CN)_6_^4−^ hydrogel electrolyte. When a temperature differential was created between the 2 ends of the thermocell, with oxidation taking place at the hot side and reduction at the cold side, a notable thermoelectric Seebeck coefficient (Se) was produced. A potential difference between the electrodes was produced by these ongoing redox reactions (Fig. 9Ai). The thermoelectric Seebeck coefficient was increased when the concentration of betaine in the hydrogel increased, as Fig. 9Aii illustrates. This improvement was explained by the redox couple’s anions binding to the betaine molecules’ cationic groups, which caused the solvation shell surrounding the redox ions to reorganize. Furthermore, a flexible thermal sensor array based on this hydrogel and carbon nanotube paper electrodes was developed for an intelligent glove (Fig. 9Aiii). When using this glove to touch toys and cups with different temperatures and shapes, an immediate voltage response was generated (Fig. 9Aiv), indicating that this thermoelectric device could sense the temperature at different locations on objects, showing great potential for wearable sensing applications. Furthermore, an I^−^/I_3_^−^ redox pair was incorporated by Wang et al. [116] into polyvinyl alcohol hydrogels in order to create an electronic skin that could sense strain and temperature in 2 modes. The hydrogel underwent redox reactions when a temperature differential was applied between its 2 ends, with oxidation taking place at the anode and reduction at the cathode. The continuous cycling of these redox reactions generated a sustained current (Fig. 9Bi). As the concentration of redox ions increased, the thermoelectric current increased, which was advantageous for the current response in subsequent sensing applications (Fig. 9Bii). Moreover, the hydrogel combined the thermogalvanic effect and the piezoresistive effect, allowing the detection of external tactile stimuli through resistance changes caused by deformation while generating power from the temperature difference. Specifically packaged hydrogel devices were used to detect facial muscle movements during chewing and finger bending actions (Fig. 9Biii). Additionally, this hydrogel thermoelectric sensor could effectively monitor the degree of neck bending and the intensity of foot movements (Fig. 9Biv).

The studies mentioned above suggest that ionic hydrogels, leveraging the ion thermodiffusion or thermogalvanic effects, offer great potential in applications such as electronic skin, monitoring of human vital signs and movements, and HMI. Nonetheless, the comparatively low energy conversion efficiency and thermoelectric figure of merit of thermoelectric hydrogels pose a major challenge. Additionally, the low electrical conductivity of gel electrolytes poses a limitation, impacting the overall performance of thermoelectric hydrogel devices. Efforts should be made to enhance conductivity without sacrificing the Seebeck coefficient. Furthermore, the packaging of gel devices is crucial for the performance of thermoelectric hydrogel sensors.

### Potentiometric mechanism

IHSSs based on piezoelectric, triboelectric, and ion diode-based mechanisms are unable to output a constant response signal in the static sensing. Therefore, the galvanic cell-based potentiometric transduction mechanism that can detect both static stimuli and low-frequency dynamic mechanical stimuli has emerged. The potentiometric transduction mechanism is a novel self-powered pressure sensing mechanism that has been studied [37,117,118]. This mechanism encodes pressure signals into continuous output electrical signals via redox reactions occurring between the 2 electrodes of the sensor. Self-powered operation and the detection of both static and dynamic stimuli are made possible by this method [119,120]. It is well known that in batteries, power supply involves external reliance on electrons and internal reliance on ions [121,122]. During the charging and discharging process, ions within the electrolyte detach from one electrode of the battery and embed into the other, thereby maintaining constant ion composition and concentration within the electrolyte [123,124]. Ionic hydrogels, as typical solid electrolytes, can maintain stable ion composition and concentration during signal transmission, making them suitable materials for the sensing layer of potentiometric sensors (Fig. 2F) [22,125,126]. External stimuli such as strain, pressure, temperature, or humidity cause the hydrogel electrolyte to deform and alter its resistance. Because of this change in resistance, the hydrogel can now operate as a self-powered pressure sensor by modifying the current signal.

**Fig. 10.**
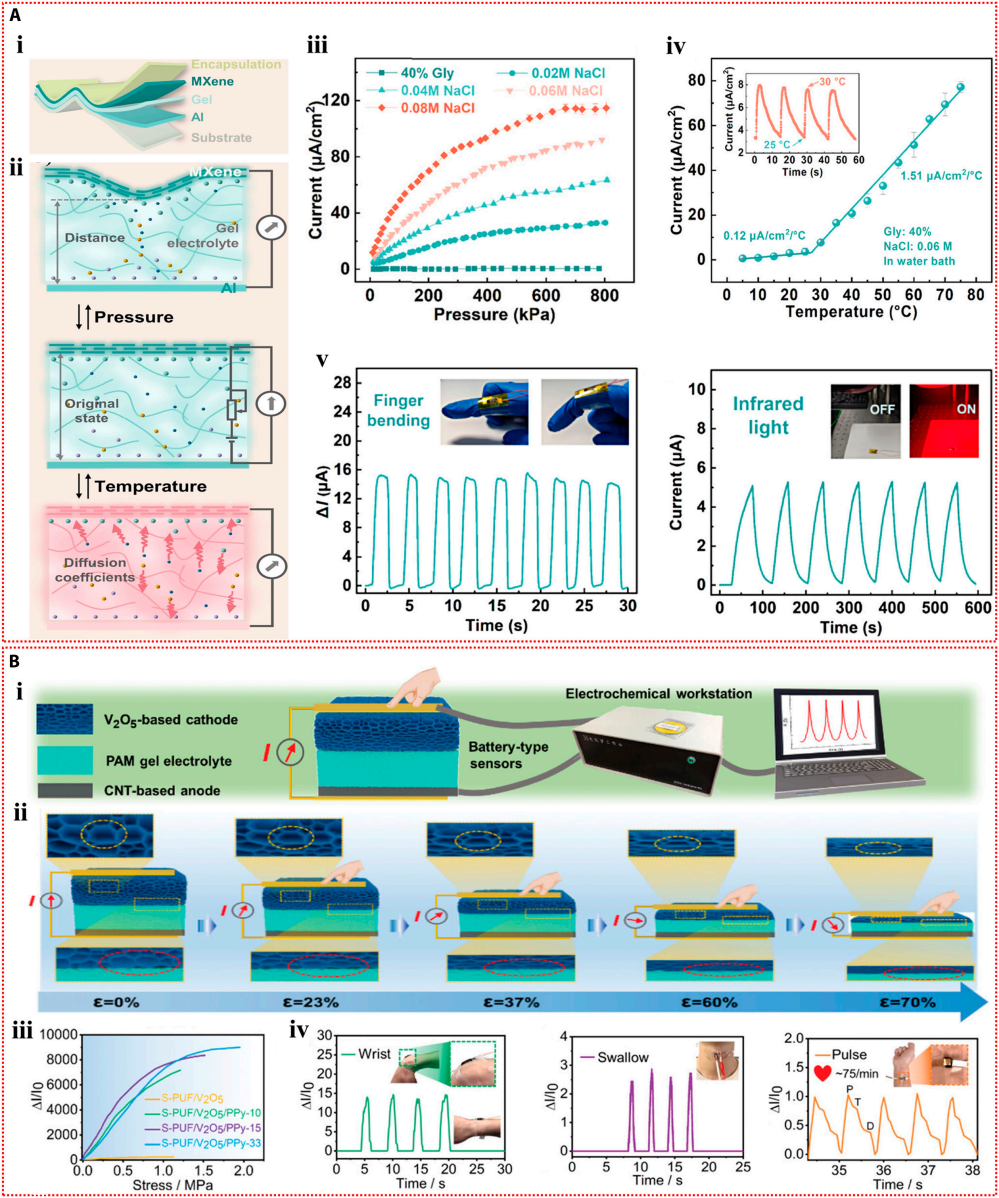
Self-powered mechanisms based on potentiometric transduction. (A) Study of potentiometric sensing performance based on the polyvinyl alcohol hydrogel containing glycerol and NaCl. (i) Schematic diagram of the structure of the potentiometric sensor. (ii) Schematic diagram of the operating principle of the potentiometric sensor under temperature and pressure stimulation. (iii) Variation in current response of the potentiometric sensor in response to different pressures utilizing the gel electrolyte with different contents of NaCl. (iv) Variation in current response of the potentiometric sensor in response to different temperatures. (v) Response signals of the potentiometric sensor in monitoring the finger bending and illumination by infrared light. Reproduced with permission from [127]. Copyright 2024, Wiley-VCH. (B) Investigation of potentiometric sensing performance based on the polyacrylamide hydrogel containing LiCl. (i) Schematic of the structural design of the potentiometric pressure sensor. (ii) Schematic diagram of the sensing mechanism of the potentiometric pressure sensor under various compressive strains. (iii) Relative current changes as a function of compressive stress of the potentiometric pressure sensor. (iv) Response signals of the potentiometric pressure sensors in monitoring the wrist bending, swallow, and pulse. Reproduced with permission from [128]. Copyright 2023, Wiley-VCH.

Using a gel made of polyvinyl alcohol, glycerol, and NaCl as the electrolyte and aluminum and MXene electrodes acting as the anode and cathode, respectively, Zou et al. [127] created a self-powered pressure–temperature dual-mode sensor (Fig. 10Ai). The sensor used the potential difference between the 2 electrodes to drive the directional movement of ions in the compressible ionic gel electrolyte, which allowed it to detect temperature and pressure in response to external stimuli (Fig. 10Aii). The self-powered sensor demonstrated a broad temperature detection range (5 to 75 °C) and a wide pressure detection range (0 to 800 kPa), as shown in Fig. 10Aiii and iv. The developed sensor showed skin-like functionality by detecting thermal stimuli from sunlight and was able to monitor physiological signals associated with human finger joint movements in real-time, indicating its promising application potential (Fig. 10Av).

Liang et al. [128] created a potentiometric self-powered pressure sensor by using an ionic hydrogel made of polyacrylamide with LiCl serving as the electrolyte. The sensor design included a composite of porous polyurethane (PU) foam, vanadium pentoxide, and polypyrrole as the cathode and pressure-sensitive layer, with porous PU foam, carbon nanotubes, and polypyrrole as the anode (Fig. 10Bi). Pressure detection was made possible by an increase in the sensor’s output current, which was caused by a decrease in the resistance of the porous electrode and gel electrolyte in response to external pressure (Fig. 10Bii). The self-powered sensor demonstrated high sensitivity and a wide pressure detection range (1.8 Pa to 1.5 MPa) (Fig. 10Biii). This sensor could detect minute pressure signals from swallowing and pulses, as well as large deformations like wrist bending (Fig. 10Biv).

Potentiometric self-powered pressure sensors provide mobility, flexibility, and self-powering by combining the capabilities of batteries and pressure sensors. They are suitable for various applications, particularly scenarios requiring long-term pressure monitoring. These sensors can be made to function as self-powered, flexible pressure sensing devices based on rechargeable batteries, which will increase their lifespan even though primary batteries may have problems with chemical energy consumption.

### Hybrid mode

One type of signal is usually produced by self-powered sensors that rely on a single energy conversion effect, which restricts their applicability in complex real-world scenarios and limits the amount of information they can provide [129,130]. Therefore, it is crucial to detect and differentiate external complex stimuli using multiple sensing principles simultaneously. Combining 2 or more self-powered mechanisms can provide new ideas for further research into revolutionizing multifunctional sensing applications, which not only compensates for the shortcomings of the single mechanism but also introduces additional functionality.

**Fig. 11.**
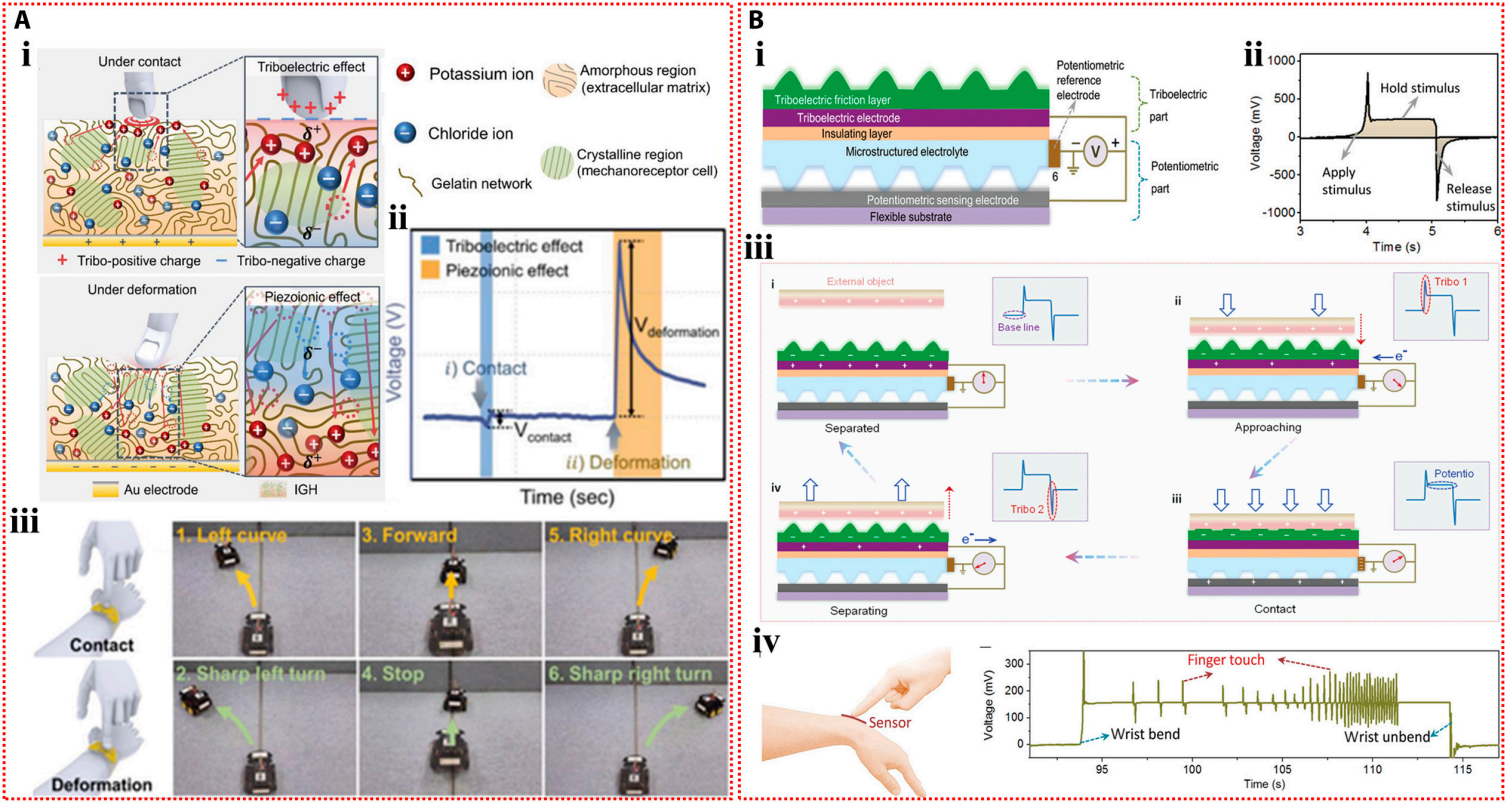
Self-powered mechanisms based on hybrid modes. (A) Investigation of triboelectric and piezoelectric performance based on the gelatin hydrogel containing potassium chloride. (i) Schematic diagram of the mechanism of the device in the hybrid modes based on triboelectric and piezoelectric effects. (ii) The output voltage of the device in the triboelectric and piezoelectric modes. (iii) Schematic of a wearable dynamically sensing communicator based on the device to control the trajectory of a miniature car. Reproduced with permission from [131]. Copyright 2021, Wiley-VCH. (B) Study of triboelectric and potentiometric performance based on the polyvinyl alcohol hydrogel containing sodium chloride and glycerol. (i) Illustration of the structural design of the mechanoreceptor. (ii) The output voltage of the device in the triboelectric and potentiometric modes. (iii) Illustration of the operating mechanism of hybrid sensing of the device. (iv) Schematic representation of the mechanoreceptor attached to the wrist and the response signals in response to bending and pressing. Reproduced with permission from [132]. Copyright 2020, Wiley-VCH.

Yoon et al. [131] developed a dynamic touch sensor using gelatin hydrogel containing potassium chloride as the functional material. This device integrated both triboelectric and piezoelectric effects to enable dynamic switching of ionic polarization states within the hydrogel (Fig. 11Ai). Ions moved to the gel surface in response to the triboelectric effect, which balanced the surface charge of the hydrogel upon contact with human skin. An opposite output signal was produced when the gel deformed due to the instantaneous ion polarization brought on by the piezoelectric effect, which redistributed the ions within the gel (Fig. 11Aii). This device was also used to develop a wearable dynamic sensing communicator that was used to control the trajectory of a small car. This demonstration (Fig. 11Aiii) illustrated how ionic hydrogel sensors may be used in human–machine communication systems. Furthermore, Wu et al. [132] combined potentiometric and triboelectric sensing principles to propose a novel self-powered mechanoreceptor using microstructured ionic hydrogel made of polyvinyl alcohol, sodium chloride, and glycerol as the solid electrolyte (Fig. 11Bi). The triboelectric sensing mode produced instantaneous signal output upon application and release of stimuli, while the potentiometric sensing mode generated a continuous and stable signal response to the stimuli (Fig. 11Bii and iii). The device fixed on the wrist could monitor static stimuli when the wrist was bent, as well as additional dynamic stimuli, and could differentiate between them based on the different signals (Fig. 11Biv).

Ionic hydrogels’ exceptional energy conversion capabilities, pliability, and biocompatibility allow them to be used in a variety of wearable applications. The application scope of conventional self-powered sensors based on ionic hydrogels can be increased by combining various energy conversion effects to create more varied sensing strategies. This approach contributes substantially to the advancement of wearable electronics.

As displayed in Table 1, we summarize the representative characteristics of the different self-powered mechanisms as well as their applications. IHSSs based on piezoelectric, triboelectric, or ionic diode mechanisms generate output signals in pulses. Thus, they are suitable for dynamic stimuli, but not for static touch. Moist-electric and thermoelectric sensors are only effective in specific environments and are more demanding in terms of environmental conditions, especially key variables such as temperature and humidity. Potentiometric sensors driven by electrochemical redox reactions can detect both static and dynamic stimuli, but at the cost of electrode corrosion. Hence, it is important to continue to develop new sensing technologies and self-powered mechanisms that can be readily applied to various situations.

**Table 1.** Comparison of different self-powered mechanisms of IHSS

Self-powered mechanisms	Energy source	Detectable stimulus	The role of ions	Advantages	Limitations	Applications
Piezoelectric	Mechanical energy: vibration, body motion, wind	Dynamic mechanical stimulus	Polarization	Good dynamic response and simple structure	Low power output, slow response, and incapable of static detection	Human motion monitoring and sound monitoring sensor
Triboelectric	Mechanical energy: vibration, body motion, ocean wave	Dynamic mechanical stimulus	Static balance	High output voltage, low material requirements, simple preparation, and high conversion efficiency	Vulnerable to environmental factors such as temperature, humidity, and impact; susceptible to wear	Wearable electronics, water height monitoring, and human–machine interface
Ionic diode	Mechanical energy/moisture energy/thermal energy	Dynamic mechanical/moist/thermal stimulus	Directed migration	Detection of low-frequency mechanical stimuli, longer current duration, and higher current density	Low power output, cumbersome preparation of bilayer gels, and unfavorable delamination	Human motion monitoring, respiration monitoring, and touch sensor
Moist-electric	Moisture energy: air moisture and human breathing	Static and slowly varying moisture stimulus	Directed migration	Extract energy directly from the atmosphere, high power output, and longer output time	Slow response, low sensitivity, and relatively cumbersome device packaging	Breathing monitors and touch pads
Thermoelectric	Thermal energy: body, the sun, instrument, facility,	Static and slowly varying thermal stimulus	Polarization/redox	More suitable for human wearability, low cost	Suitable for steady-state temperature monitoring, low energy conversion efficiency, and low power output	Touch sensor, wearable electronics, fire alarm, and environment monitoring
Potentiometric	Mechanical energy/thermal energy/moisture energy	Static and slowly varying mechanical/moist/thermal stimulus	Redox	Long-term monitoring, high power output, and capable of static detection	Chemical energy consumption issues	Human motion monitoring, respiration monitoring, and touch sensor

## Structural Engineering

Ionic hydrogels offer unique advantages in structural and performance design due to their excellent flexibility, ease of preparation, and processing. Researchers have focused on carefully crafting the ionic hydrogel sensing layer in order to improve sensing performance and increase the application range of IHSS devices. A number of important features, such as sensitivity, sensing range, response time, stability, and multifunctionality, are intended to be improved by this design optimization. In order to provide more design ideas for the future development of IHSS devices, we focus on the microstructure design of IHSS in this subsection as well as the performance design, which includes mechanical properties, self-healing design, and environmental stability.

### Substrate materials

IHSS devices are built on a substrate material, which also directly affects the devices’ functionality and range of applications. For IHSS devices, the substrate material should be lightweight, easy to process, readily available, stretchable/compressible, chemically stable, and thermally stable. Selecting an appropriate substrate material based on the corresponding performance and application requirements can enhance the overall performance of IHSS devices and meet the demands of various application environments.

Polymer materials are the most commonly used substrates in IHSS devices due to their superior flexibility, electrical insulation, thermal stability, and formability. Polyimide (PI), polyethylene terephthalate (PET), polycarbonate (PC), polybutylene terephthalate (PBT), polyethersulfone (PES), and polypropylene (PP) are examples of common polymer materials [133–138]. Furthermore, silicone rubber, PU, silicone–butadiene rubber (SBR), thermoplastic elastomers (TPEs), polydimethylsiloxane (PDMS), and silicone rubber are all excellent options for IHSS substrates [41,139–141]. These materials can be directly involved in the functional processes of the sensor, in addition to acting as carriers for the sensor electrodes and sensing layers or encasing the device. Furthermore, due to their biocompatibility, conformability, low cost, and ease of availability, paper and fabric can be used as IHSS substrate materials [142–145]. Additionally, some semiconductor materials can also be used as substrates, although they tend to be more rigid [146]. Choosing the right substrate material is crucial for manufacturing IHSS devices. In practical applications, selecting appropriate materials must be guided by the specific needs and performance requirements of the sensor. This ensures that the sensor meets the desired criteria for functionality, durability, and efficiency in its intended application. It is also possible to further optimize performance through modifications or by combining with other materials, thereby achieving high-performance IHSS devices.

### Electrode materials

As research into sensors deepens, the importance of electrodes, a necessary component of self-powered sensors, becomes increasingly prominent. The stability and conductivity of the electrodes directly affect the functionality of the sensor. Thus, research is progressing from manufacturing stable electrodes to seeking electrodes with superior performance. The selection of electrodes for self-powered sensors generally requires consideration of several factors, including material conductivity, chemical stability, mechanical strength, and sensitivity [147,148]. Generally, common electrode materials are divided into 2 categories: inert electrodes and active electrodes. When designing self-powered sensors, suitable inert electrodes, active electrodes, or a combination of both can be selected based on the specific sensing mechanism and application requirements, aiming to achieve the desired sensing performance and stability.

### Inert electrode materials

Inert electrodes typically refer to electrode materials that do not greatly affect electrochemical reactions. These electrodes are mainly used in sensors to provide a stable electrode reaction environment without participating in the sensing process itself. The selection of inert electrodes usually considers factors such as conductivity, chemical stability, and mechanical strength. The most widely used inert electrodes are carbon-based materials like graphene, carbon nanotubes, and MXene, as well as metal electrodes like gold and platinum [36,104,149]. Additionally, stretchable flexible composite hydrogels formed by incorporating ions or carbon-based materials or conductive polymers into the hydrogel can also serve as inert electrodes [84,150,151].

### Active electrode materials

Active electrodes refer to electrode materials that undergo redox reactions during electrochemical processes. These electrodes can directly participate in the sensing process, achieving sensing functions through reactions with the ionic hydrogel sensing layer [152,153]. The selection of active electrodes needs to consider their reactivity with the active layer and factors such as sensor sensitivity and selectivity [154,155]. The most common active electrodes include metal electrodes like aluminum, iron, copper, zinc, and nickel. Additionally, Ag/AgCl electrodes, which can undergo redox reactions, also fall into the category of active electrodes [37,156].

Although metal-based materials offer excellent conductivity, their lack of stretchability limits their potential in flexible and stretchable applications. On the other hand, nonmetal-based materials typically have good stretchability, making them better suited for enhancing the comfort and wearability of flexible self-powered sensors. Furthermore, the target electrode’s structure and form can be modified to suit particular application requirements. However, selecting the appropriate electrode material requires a comprehensive evaluation of the specific sensing mechanism and application requirements to ensure optimal overall performance.

### Structure and performance design

With the continuous advancement and intelligent development of flexible sensors, the structural design and material properties of IHSS are becoming increasingly diverse. The structure of IHSS devices, based on ion transport regulation, primarily consists of 3 parts: the substrate, the electrode, and the ionic hydrogel sensing layer. This section reviews notable advancements in the structural and performance design of IHSS in recent years. The goal is to explore the various factors and emerging trends that should be considered in future sensor design to further enhance their effectiveness and applicability.

### Microstructure design

Sensitivity is one of the important indicators for evaluating the performance of the sensor. It refers to the sensitivity of the sensor’s output response to changes in the measured physical quantity. The accuracy and stability of the measurement can be visualized by the sensitivity. The development of high sensitivity is an area that has been studied thoroughly as high sensitivity is the key to detecting weak signals. The sensitivity of the self-powered sensor can be expressed as 𝑆 = 𝛿(∆𝑋/𝑋_0_)/𝛿Z. 𝑋_0_ represents the value of the initial electrical signal of the sensor, such as current and voltage; ∆𝑋 represents the relative variation of the electrical signal; Z represents the external stimuli such as pressure, humidity, or temperature. The sensitivity of hydrogel sensors is generally low, which is a key issue restricting their development and application. In addition to maximizing the composition of ionic hydrogels, creating biomimetic surface microstructures can be a useful technique to increase IHSS sensitivity and decrease the detection limit. Micro-nano fabrication techniques can create various micro-nano structures such as pyramids, cones, and columns to maximize the efficient contact region between sensing layer and electrodes, thereby enhancing sensor performance [157–161]. The design of microstructures is more commonly seen in pressure-based self-powered sensors. Additionally, chemical modification strategies such as nanoparticle filling, surface fluorination, and chemical functionalization have been developed to create surfaces with various unique properties, thus achieving improved sensing performance and multifunctionality [162–164].

**Fig. 12.**
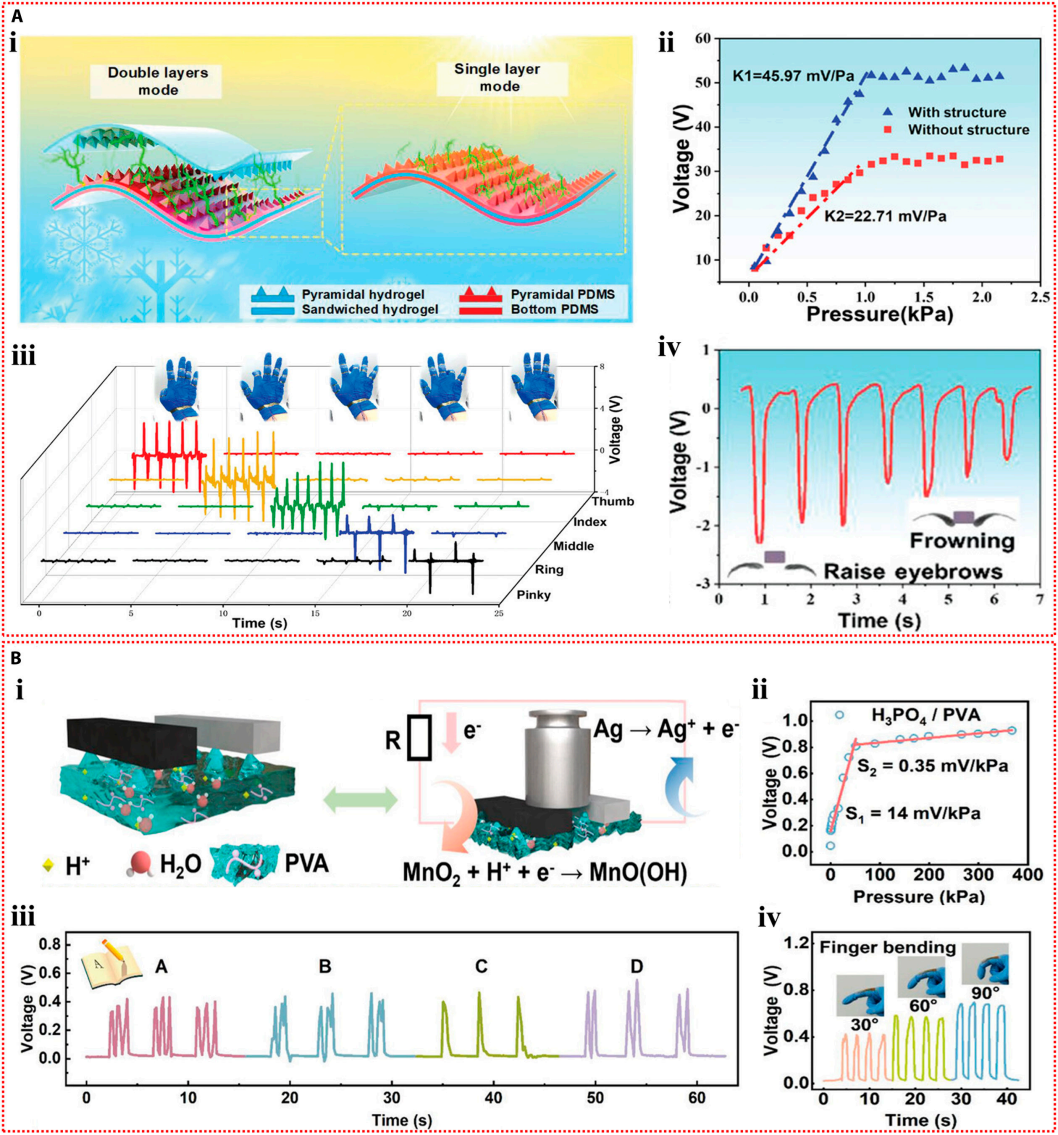
Microstructure design of IHSS devices. (A) Investigation of the microstructure design of triboelectric sensor based on the polyacrylamide/carrageenan hydrogel containing lithium bromide. (i) Schematic representation of the micro-pyramidal structure design of the device. (ii) Output voltage variations at different pressures in the single-electrode mode for devices with and without structural design. (iii) Response signals of the triboelectric sensors fixed on 5 fingers in response to the finger joint motion. (iv) Response signals of the triboelectric sensor in response to the eyebrow motion. Reproduced with permission from [75]. Copyright 2022, Wiley-VCH. (B) Study of the microstructure design of potentiometric sensor based on the polyvinyl alcohol hydrogel containing phosphoric acid. (i) Schematic diagram of the structure and working principle of the sensor. (ii) Sensitivity curve of the potentiometric sensor at different pressures. (iii) The response signals of the sensor to writing various letters. (iv) The response signals of the sensor to finger bending. Reproduced with permission from [165] . Copyright 2024, Wiley-VCH.

By adding lithium bromide to a dual-network hydrogel made of polyacrylamide and carrageenan, Tao et al. [75] created an ionic hydrogel with micro-pyramidal structures (Fig. 12Ai). The triboelectric sensor based on this ionic hydrogel dramatically improved its pressure sensing performance because of the presence of the micro-pyramidal structure, which allowed it to achieve a high sensitivity of 45.97 mV Pa^−1^, which was twice the sensitivity of nonstructured sensors (Fig. 12Aii). This self-powered sensor not only exhibited excellent sensitivity in monitoring human finger joint movements but also had a good perception of subtle eyebrow movements (Fig. 12Aiii and iv). Yang et al. [165] used polyvinyl alcohol and phosphoric acid to create an ionic hydrogel with a microstructured surface resembling sandpaper. A potentiometric sensor was then assembled with this ionic hydrogel as the electrolyte, and MnO₂ and silver were used as the electrodes (Fig. 12Bi). The sandpaper-like microstructure design of the hydrogel substantially improved the device’s sensitivity under low pressure (Fig. 12Bii). The self-powered sensor could accurately recognize different written letters and distinguish various degrees of finger bending (Fig. 12Biii and iv).

Furthermore, Liang et al. [155] used a mesh molding method to prepare an ionic hydrogel electrolyte with a microstructured surface, composed of polyvinyl alcohol, NaCl, and glycerol. The microstructured hydrogel electrolyte was sandwiched between zinc and carbon electrodes to form a typical potentiometric sensor. The hydrogel’s microstructured surface steadily expanded the electrode–electrolyte interface’s contact area when the apparatus was compressed. The device’s internal impedance quickly decreased as a result of the contact area expansion, raising the potential difference between the electrodes. This led to high sensitivity (234.15 mV N^−1^). The self-powered sensor had a good perception of vocal cord vibrations, accurately distinguishing the pronunciations of different words. Additionally, the sensor could precisely monitor human pulse vibrations and walking states, indicating a wide range of applications.

### Environmental stability design

Typically, hydrogels are moist, soft materials that contain a large amount of water. Nevertheless, hydrogels are not very adaptable to harsh environments because of their high water content [166]. Conventional hydrogels have a tendency to freeze below 0 °C, which causes them to harden and lose some of their mechanical qualities, conductivity, adhesion, and flexibility [167,168]. In dry environments, hydrogels are prone to severe water loss, leading to network structure collapse and deformation [169,170]. For self-powered sensors based on ionic hydrogels, freezing or drying of the hydrogel severely affects internal ion migration, thereby adversely impacting sensor performance. Therefore, it is crucial to maintain the hydrogel’s wet and soft activity under extreme conditions, endowing it with environmental adaptability. The most common approach to enhancing the environmental stability of ionic hydrogels involves introducing organic solvents or salts (ions) to modify the composition of free water [47,171]. Furthermore, although this approach is more involved, it is possible to further enhance environmental stability by modifying the gel network or designing it to enhance the interaction between water molecules and the gel matrix [172,173]. Of course, incorporating ionic liquids into the gel can also enhance its environmental stability [174,175], but the resulting gels are classified as ionogels rather than hydrogels and are therefore not discussed here. The antifreeze and moisturizing properties of ionic hydrogels are crucial for the normal operation, stable performance, measurement accuracy, and lifespan of self-powered sensors, ensuring reliable performance under various environmental conditions.

**Fig. 13.**
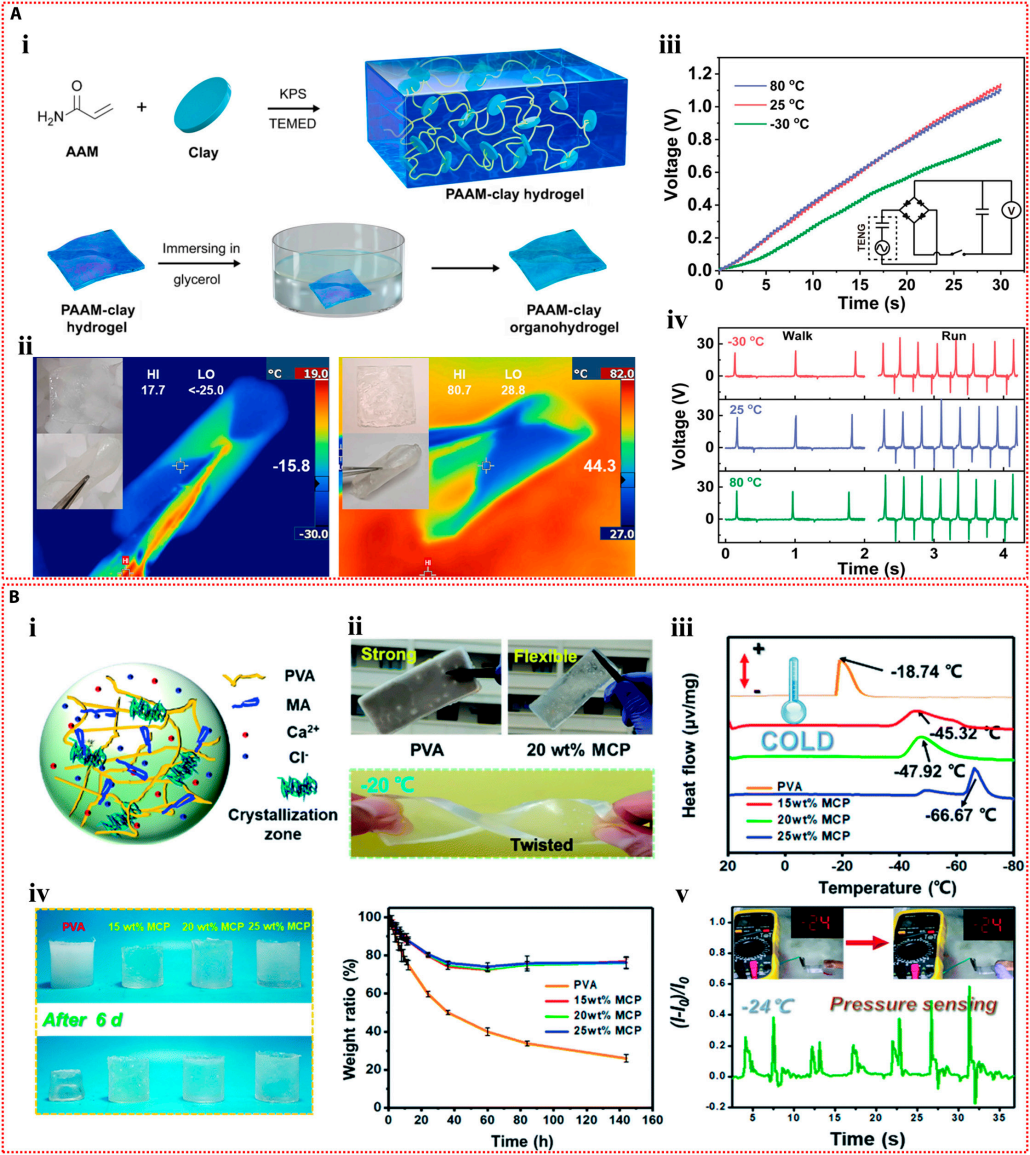
Environmental stability design of IHSS devices. (A) Investigation of the environmental stability design of triboelectric sensor based on the polyacrylamide hydrogel containing clay and glycerol. (i) Schematic diagram of the preparation of the ionic organohydrogel. (ii) Thermal images of the ionic organohydrogel at −30 and 80 °C, respectively. (iii) Voltage curves of a capacitor being charged by the ionic organohydrogel-based TENGs at different temperatures. (iv) The response signals of the triboelectric sensor to the human body in walking and running at different temperatures. Reproduced with permission from [178]. Copyright 2021, Elsevier. (B) Study of the environmental stability design of potentiometric sensor based on the polyvinyl alcohol hydrogel containing calcium chloride and malic acid. (i) Schematic diagram of the composition of the ionic hydrogel. (ii) Photos of the pure hydrogel and ionic hydrogel after freezing at −20 °C for 48 h, respectively. (iii) Differential scanning calorimetry (DSC) results of the pure hydrogel and ionic hydrogels from 20 to −80 °C. (iv) Photos and weight changes of the pure hydrogel and ionic hydrogels after 6 d of storage at 25 °C and 60 RH% (RH denotes relative humidity). (v) The sensing signals of the potentiometric pressure sensor at −24 °C. Reproduced with permission from [181]. Copyright 2021, Royal Society of Chemistry.

Typically, introducing organic solvents into ionic hydrogels can effectively enhance their environmental stability. The polar groups of organic solvents are able to create numerous hydrogen bonds with the water molecules within hydrogels, weakening the interactions between “free water” molecules, thus achieving antifreeze and moisturizing effects [176,177]. Huang et al. [178] first prepared an ionic hydrogel using acrylamide and clay as raw materials through polymerization and then immersed the hydrogel in glycerol for solvent exchange, resulting in an environmentally stable ionic organohydrogel (Fig. 13Ai). Because of the hydrogen bonds that formed between the glycerol and water molecules within the gel, this ionic organohydrogel maintained excellent flexibility over a wide temperature range, from −30 to 80 °C (Fig. 13Aii). Moreover, this ionic organohydrogel could be used as an electrode in a TENG. The electrical energy generated at different temperatures could be stored in a capacitor after being converted from AC to DC by an inverter (Fig. 13Aiii). Additionally, the device functioned as a self-powered sensor, capable of detecting human activities such as walking and running under various temperature conditions (Fig. 13Aiv).

Adding a high content of salt to hydrogels can also enhance their environmental stability. Salt ions tightly bind to the free water in the gel in the form of hydrated water, dramatically improving the environmental adaptability of the hydrogel [179,180]. Wang et al. [181] introduced calcium chloride and malic acid into polyvinyl alcohol (PVA) hydrogels to prepare a stretchable ionic hydrogel (Fig. 13Bi). The ionic hydrogel maintained its flexibility at −20 °C, whereas pure PVA hydrogel freezes at that temperature (Fig. 13Bii). Further analysis using differential scanning calorimetry revealed that the ionic hydrogel with the best performance had a freezing point of −47.92 °C, indicating good antifreeze qualities (Fig. 13Biii). The ionic hydrogel also exhibited outstanding moisturizing performance (Fig. 13Biv). The superior environmental stability of this ionic hydrogel was attributed to the interaction between Ca^2+^ and the free water in the hydrogel. Additionally, a potentiometric self-powered strain sensor was constructed employing this ionic hydrogel as an electrolyte, with zinc and copper sheets as electrodes. This self-powered sensor demonstrated stable perception of finger pressing even at low temperatures (Fig. 13Bv).

### Mechanical property design

Soft ionic hydrogels, as promising candidates for flexible self-powered sensors, indeed have many advantages. However, they also face several challenges, one of which is the relatively low mechanical performance and limited stretchability of traditional hydrogels [182,183]. This results in inadequate adaptability to 3D surfaces and dynamic environments. To address these challenges, researchers have devised new strategies to synthesize novel hydrogels with enhanced mechanical properties, which in turn improves the overall performance of sensors.

**Fig. 14.**
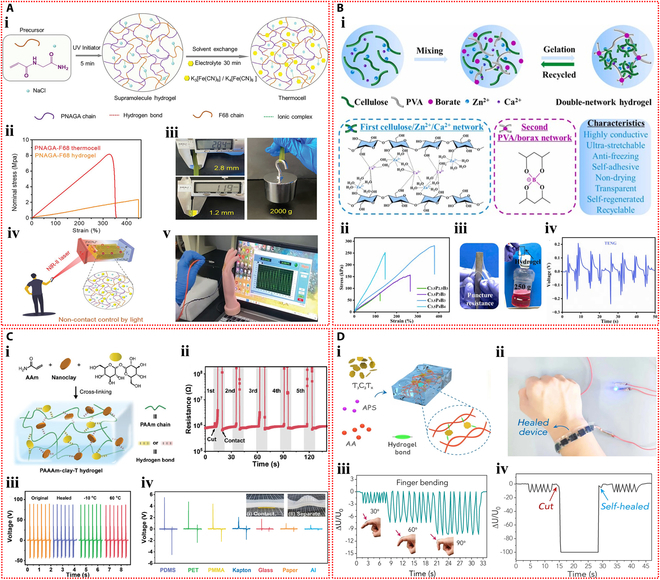
Mechanical properties and self-healing design of IHSS devices. (A) Study of the mechanical property design of thermoelectric sensor based on the poly(N-acryloyl glycinamide) hydrogel containing diacrylate-capped Pluronic F68 crosslinker, Fe(CN)_6_^3−^/Fe(CN)_6_^4−^ redox couple, and sodium chloride. (i) Illustration of the preparation of the ionic hydrogel thermocell. (ii) Stress–strain curves of pure hydrogel and ionic hydrogel thermocell. (iii) Photographs of the thermocell used for moving heavy loads. (iv) Illustration of the non-contact power supply for the thermocell remote-controlled by light. (v) Non-contact information transmission of the self-powered ionic skin sensor remote-controlled by light. Reproduced with permission from [184]. Copyright 2023, Wiley-VCH. (B) Investigation of the mechanical property design of triboelectric sensor based on the PVA–borax hydrogel containing zinc chloride, calcium chloride, and cellulose. (i) Schematic of the preparation and characteristics of the double-network ionic hydrogel. (ii) Stress–strain curves of the ionic hydrogels with different contents of PVA and borax. (iii) Pictures of the puncture resistance of the ionic hydrogel and its ability to lift a 250-g bottle. (iv) The response signals generated by the triboelectric sensor to the written letters. Reproduced with permission from [185]. Copyright 2024, Elsevier. (C) Study of the self-healing design of triboelectric sensor based on the polyacrylamide hydrogel containing clay and trehalose. (i) Schematic of the composition of the ionic hydrogel. (ii) Resistance changes during repeated cutting and contacting of the ionic hydrogel. (iii) The voltage signals of the sensor before and after the self-healing. (iv) Voltage signals of the sensor in contact with different materials. Reproduced with permission from [195]. Copyright 2023, Wiley-VCH. (D) Investigation of the self-healing design of triboelectric sensor based on the polyacrylic acid hydrogel containing MXene nanosheets, Fe(CN)_6_^3−^/Fe(CN)_6_^4−^ redox couple, and guanidinium chloride. (i) Schematic of the composition of the ionic hydrogel. (ii) Photograph of the self-healing strip thermoelectric device array powering an LED bulb by harvesting body heat. (iii) The response signals of the triboelectric sensor to finger bending. (iv) Voltage changes of the triboelectric sensor before and after the cut-healing process. Reproduced with permission from [196]. Copyright 2023, Springer.

Shi et al. [184] introduced a macromolecular crosslinker, diacrylate-capped Pluronic F68, and the Fe(CN)_6_^3−^/Fe(CN)_6_^4−^ redox couple into poly(N-acryloyl glycinamide) hydrogels to prepare a dual hydrogen bond-enhanced ionic hydrogel (Fig. 14Ai). The stress–strain curves in Fig. 14Aii demonstrated that the ionic hydrogel exhibited high tensile strength and a substantial Young’s modulus. The elongated hydrogel was capable of supporting a weight of 2,000 g without breaking and could endure deformations (Fig. 14Aiii). Furthermore, the thermoelectric cell based on this ionic hydrogel could generate electricity by creating a temperature gradient through remote optical control (Fig. 14Aiv). This thermocouple was used as a self-powered smart skin sensor, enabling noncontact information transmission controlled by light (Fig. 14Av). Wang et al. [185] synthesized the first network by introducing zinc chloride and calcium chloride into rigid cellulose hydrogels, and then formed the second network through the crosslinking reaction between PVA and borax, thereby preparing a double-network ionic hydrogel with good mechanical properties (Fig. 14Bi). The hydrogel with multiple crosslinked networks exhibited excellent mechanical properties, showing outstanding puncture resistance and fracture toughness (Fig. 14Bii and iii). Additionally, the triboelectric tactile sensor based on this ionic hydrogel could recognize different letters and handwriting (Fig. 14Biv).

By using these novel approaches, hydrogels’ mechanical characteristics are improved, which makes them more suited for flexible self-powered sensors and improves the sensors’ overall performance and dependability. More innovative techniques and materials are anticipated to emerge in the future as a result of ongoing technological innovation and progress, propelling further growth and developments in the field of flexible self-powered sensors.

### Self-healing design

Human skin has the ability to self-repair after injury, a self-healing characteristic that has inspired interest in mimicking this capability in the field of sensors [186,187]. With advancements in self-healing ionic materials, such as hydrogels and ionic polymers/gels, researchers have shown a growing interest in developing next-generation sensors with self-healing capabilities. There are generally 2 main ways that polymer materials can become self-healing. The first method involves dynamic covalent crosslinking based on bond breaking and reformation, which requires external stimuli or healing agents to trigger the healing process [188,189]. The second method utilizes noncovalent interactions such as hydrogen bonding, ionic crosslinking, π–π interactions, and host–guest interactions [190–192]. These noncovalent interactions can more effectively achieve autonomous self-healing without the need for external stimuli [193,194]. Researchers are working to develop more efficient noncovalent interaction techniques to accomplish autonomous self-healing in environmental settings, emulating human skin. This self-healing ability is crucial for developing more sustainable, stable, and long-lasting sensors, reducing maintenance costs and time, and thereby advancing sensor technology and applications.

By adding trehalose and sodium ion-containing clay to polyacrylamide hydrogel, Dai et al. [195] created an ionic hydrogel with amazing mechanical and self-healing properties (Fig. 14Ci). The ionic hydrogel’s remarkable self-healing performance was demonstrated by its ability to retain its resistance even after numerous cutting and self-healing cycles, which was ascribed to the hydrogen bonding interactions among the 3 raw materials (Fig. 14Cii). Moreover, self-powered sensing was made possible by using this ionic hydrogel as the electrode material in a triboelectric sensor. Following the self-healing process, the sensor material showed almost no reduction in output voltage peaks because of the self-healing characteristics of both the charged and electrode layers (Fig. 14Ciii). Integrated into a soft robot, the sensor generated different output signals when in contact with different materials, indicating that the triboelectric sensor could be integrated with robots to sense materials (Fig. 14Civ).

Moreover, using acrylic acid as the monomer, MXene nanosheets as the crosslinker and promoter, a Fe(CN)₆^3−^/Fe(CN)₆^4−^ redox pair, and guanidinium chloride as the ion source, Lu et al. [196] created a self-healing ionic hydrogel (Fig. 14Di). The hydrogel’s ability to heal itself was greatly improved by the multiple hydrogen bonds that were created between the terminal groups of MXene and the carboxyl groups on the polyacrylic acid chains. After being cut and allowing it to heal itself, the thermoelectric array based on this ionic hydrogel could still use body temperature to illuminate an LED bulb (Fig. 14Dii). Furthermore, the thermoelectric device based on this ionic hydrogel could serve as a self-powered sensor to monitor human finger movements (Fig. 14Diii). The device exhibited repeatable electrical signals even after undergoing cutting and healing processes, demonstrating excellent recovery (Fig. 14Div).

### Other property designs

**Fig. 15.**
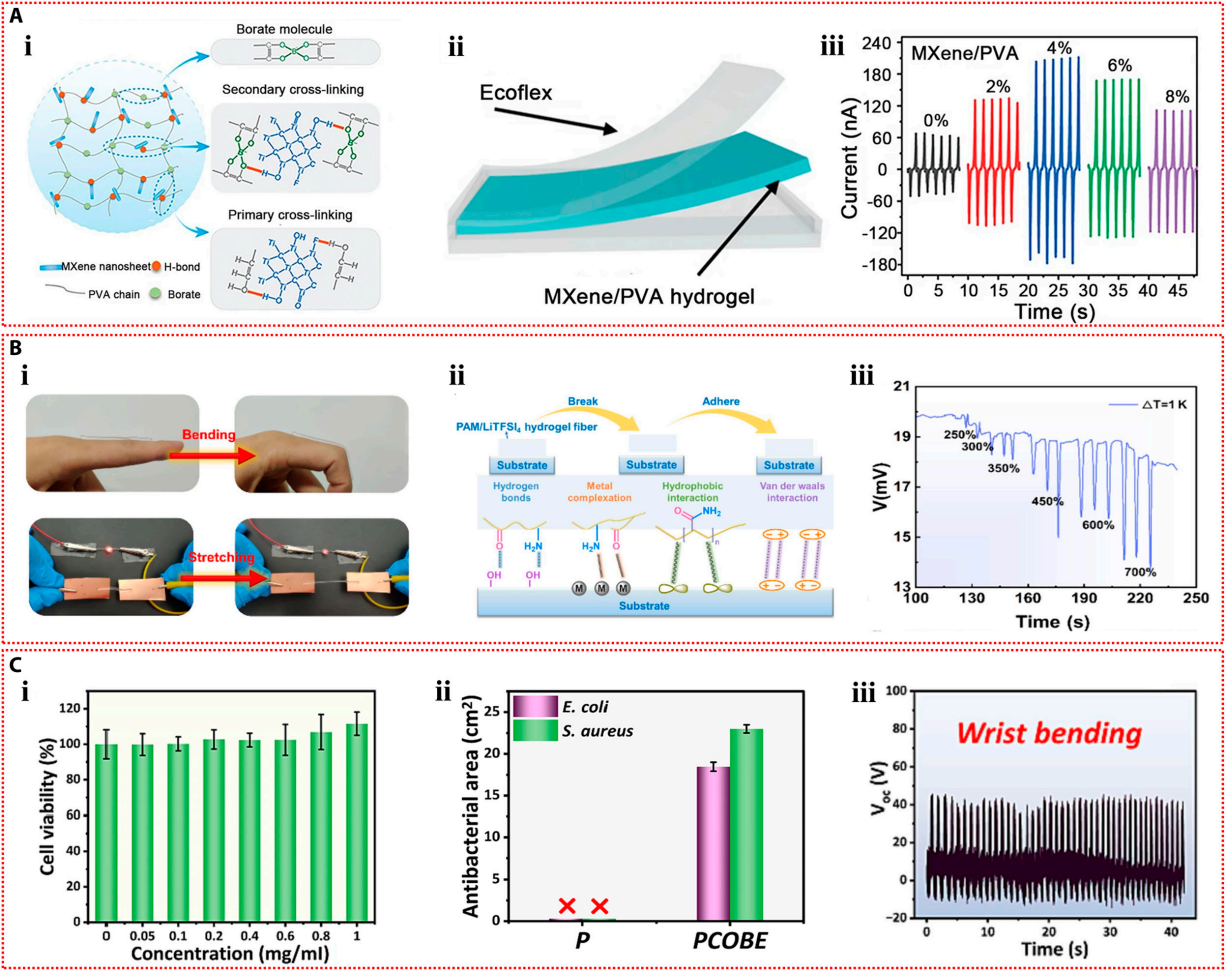
Conductivity, self-adhesion, and biocompatibility design of IHSS devices. (A) Investigation of the conductivity design of triboelectric sensor based on the polyvinyl alcohol hydrogel containing sodium tetraborate and MXene. (i) Schematic of the composition of the ionic hydrogel. (ii) schematic structure of the triboelectric generator. (iii) Short-circuit current of the triboelectric generator for different doping concentrations of MXene nanosheets. Reproduced with permission from [197]. Copyright 2021, Wiley-VCH. (B) Study of the self-adhesion design of thermoelectric sensor based on the polyacrylamide hydrogel containing lithium bis(trifluoromethane) sulfonimide. (i) Photographs of the hydrogel fiber conformally adhered to the index finger and self-adhered to the Cu sheet without falling off. (ii) Schematic diagram of the self-adhesion mechanism. (iii) Voltage signals of the thermoelectric sensor at different strains. Reproduced with permission from [199]. Copyright 2024, Elsevier. (C) Investigation of the biocompatibility design of triboelectric sensor based on the Zr^4+^ crosslinked polyacrylic acid hydrogel containing collagen, oxidized hyaluronic acid, silver nanoparticles, and ethylene glycol. (i) The biocompatibility test. (ii) Antibacterial performance of the hydrogel. (iii) The response signals of the triboelectric sensor to wrist bending. Reproduced with permission from [201]. Copyright 2023, Elsevier.

Since hydrogel is commonly used as an active or electrode layer in flexible sensors, its conductivity is an important material property. One of the unique advantages of hydrogels over other sensing materials is the adjustable conductivity. It is easy to introduce conductive additives into hydrogel precursors to tailor the conductivity. For ionic hydrogels, the presence of internal ions already provides good conductivity. Moreover, for some specific mechanisms or applications, the conductivity of the ionic hydrogel should be further optimized to ensure a more excellent output response and sensing capability of the sensor. Typically, common strategies to improve the conductivity of ionic hydrogel include the introduction of dopants such as conductive nanofillers (e.g., carbon nanomaterials and metal nanomaterials) or conductive polymers [e.g., polyaniline, poly(3,4-ethylenedioxythiophene), and polypyrrole] into the gel matrix. This is done by generating conductive transport channels within the hydrogel to achieve an increase in conductivity. For example, Luo et al. [197] introduced MXene nanosheets into the polyvinyl alcohol hydrogel containing sodium tetraborate to prepare a highly conductive hydrogel (Fig. 15Ai). The conductivity of PVA hydrogels was greatly improved by the dual cross-linking of MXene and borates. The hydrogel was encapsulated in Ecoflex silicone rubber to assemble the single-electrode triboelectric generator (Fig. 15Aii). Based on the flow vibrational potential model, the structure of MXene nanosheets resembles a water-filled microchannel, which facilitates the transport of positive ions in the hydrogel after tribo-charging, thus improving the output performance (Fig. 15Aiii). The device shows great potential for applications in wearable motion monitoring and action recognition.

Furthermore, self-adhesion is important for IHSS in wearable applications and biologically relevant applications. Traditional wearable sensors require additional tapes or bandages to secure them to the body, causing increased discomfort in wearing them. Adhesion ensures a mechanical match between the skin and the sensor, enhancing the stability and durability of the sensor in practical applications, which is one of the prerequisites for wearable sensors to achieve a sensitive and stable signal output. Based on reversible physical interactions (e.g., hydrogen bonding interactions, electrostatic interactions, hydrophobic associations, and π–π interactions), hydrogels can form strong interfacial interactions with a wide variety of substrates, thereby promoting adhesion [198]. For instance, Dai et al. [199] developed an ionic thermoelectric hydrogel fiber with excellent self-adhesion using polyacrylamide and lithium bis(trifluoromethane) sulfonimide as raw materials. Based on the strong self-adhesive ability, the hydrogel fiber would not slide or delaminate from the skin interface during the repeated bending of the finger (Fig. 15Bi). Also, the hydrogel fiber, which were self-adhesive to the 2 copper sheets, could be stretched to connect to the circuit and light up a LED. The unique self-adhesion of hydrogel fiber is due to the numerous reversible physical bonds formed between the polyacrylamide polymer chains and LiTFSI of the gel and the substrates, such as hydrogen bonding, van der Waals forces, metal complexation, and hydrophobic interactions (Fig. 15Bii). Wearable sensors based on the hydrogel fiber can achieve self-powered sensing by generating a thermal voltage, producing a good response to different strains (Fig. 15Biii).

Besides, biocompatibility is an essential requirement for IHSS in wearable and bio-related applications. Prolonged direct contact between hydrogel and human skin can lead to conditions such as sensitization and redness, which greatly increases the frequency of replacement and limits the application of IHSS. Therefore, it is important to enhance the biocompatibility of ionic hydrogels. Currently, the common strategies to obtain good biocompatibility are the introduction of biomass macromolecules (starch, collagen, silk, chitosan, alginate, gelatin, and cellulose) into polymer hydrogels [200]. For example, Song et al. [201] introduced collagen, oxidized hyaluronic acid, silver nanoparticles, and ethylene glycol into the Zr^4+^ crosslinked polyacrylic acid hydrogel to prepare an organohydrogel with good biocompatibility. HeLa cells were cultured using extraction solutions with different concentrations of gels, and the results showed that the survival rates of the cells were more than 95% (Fig. 15Ci). Moreover, the presence of silver nanoparticles conferred good antibacterial capacity to the gel (Fig. 15Cii). The triboelectric sensor based on the gel can be applied as a wearable sensor for stabilized monitoring of the bending of the human wrist (Fig. 15Ciii).

The summary and comparison of working mechanisms, materials, electrode, structures, sensing performance, and characteristics of recently reported IHSSs is summarized in detail as displayed in Table 2. Generally, IHSS can be categorized into piezoelectric IHSS, triboelectric IHSS, ionic diode IHSS, moist-electric IHSS, thermoelectric IHSS, and potentiometric IHSS depending on the sensing mechanisms and modes. First, the selection of hydrogel raw materials plays an important role in the sensing performance and functional properties of IHSS. The selection of raw materials and the design of specific systems should be based on the desired performance and specific application requirements to achieve better performance. In addition, the sensing performance of IHSS, including sensitivity, response time, and response range, can be substantially improved through the specific structural design. Furthermore, it is also important to select the suitable electrodes to achieve the desired sensing performance and stability. In particular, owing to the recent attention and rapid development of IHSS, it is gratifying to see that the new sensing modality offers great advantages in terms of high sensitivity, fast response, wide response range, and dynamic/static response, which can lay the foundation for further exploitation of self-powered sensors with excellent sensing characteristics.

**Table 2.** Summary and comparison of IHSS with different mechanisms and materials

Mechanisms	Compositions	Electrode	Structural engineering	Sensing information	Sensitivity	Response range	Response time	Characteristics	Ref.
Piezoelectric	Poly(AA-co- AAm)/NaCl	Au	Array	Pressure	8 μV kPa^−1^	<360 kPa	>30 ms	High-charge density	[58]
Piezoelectric	PAM/PAMPS/SnSe/K_3_Fe(CN)_6_	Ag nanowires	Addition of nanomaterials	Pressure	1,780 nV Pa^−1^, −7.21 nA Pa^−1^.	—	Voltage: 6–8 ms; current: 428 ms	High piezoionic coefficient	[59]
Piezoelectric	Poly(AAm-co-SPA)	Au	3D printing of stacked ionic assemblies	Pressure	0.95 mV kPa^−1^	<150 kPa	—	Use in object mapping, recognition, and localization	[60]
Triboelectric	PAAm/LiCl	LiCl hydrogel	Sandwich architecture	Pressure	0.013 kPa^−1^	<446.2 kPa	—	Biomechanical energy harvesting and tactile sensing	[72]
Triboelectric	PVA/NaCl	NaCl hydrogel	Pocket structure with surface microstructure	Pressure	1.95 V kPa^−1^, 0.5 V kPa^−1^	0–11.28, 11.28–56.63 kPa	—	Greenness, stretchability, and dangerous driving behavior monitoring	[73]
Triboelectric	Carrageenan/PAAm/KCl/LiBr	KCl/LiBr hydrogel	Micro-pyramid-patterned structure	Pressure	45.97 mV Pa^−1^	50 Pa–9 kPa	~20 ms	Remarkable flexibility, good transparency, ultrasensitivity, and environmental stability	[75]
Ionic diode	Agar/PSS/PDACl /MXene/EG	Carbon cloth	Sandwich architecture	Pressure/moisture	Pressure: 0.013 V kPa^−1^/0.93 mV/RH%	<7.30 kPa/60–90% RH	—	Environmental stability and dual-stimulus responsiveness	[83]
Ionic diode	PAM/MSA/MCS	NaCl hydrogel	Sandwich architecture	Pressure/moisture/temperature/salinity	679.62, 35.16 mV MPa^−1^	50 Pa–0.5 MPa, 0.1–0.5 MPa	—	Entirely composed of ionic conductors and multifunctionality	[84]
Ionic diode	Agar/PAAm/PSS/PDACl/EG	Ag/AgCl	Sandwich architecture	Pressure/moisture	—	13–85% RH	—	Stretchability, environmental stability, and multimodal sensation	[85]
Moist-electric	Poly(AAm-co-AMPS)/LiCl	Ag/Pt	Asymmetric hygroscopic structure	Moisture	—	30–80% RH	—	High performance and high stretchability	[39]
Moist-electric	AG/PAM/LiCl/CBs@fabric	Cu	Sandwich architecture	Pressure	—	—	—	Stretchability and weight identification	[99]
Thermoelectric	PQ-10/NaOH	Graphite	Array	Temperature	2.7 mV K^−1^	281.15– 313.15 K	—	Ultrasensitivity and dual-mode sensing	[113]
Thermoelectric	Gelatin/betaine/K_3_Fe(CN)_6_/K_4_Fe(CN)_6_	Carbon nanotube papers	Array	Temperature	2.2 mV K^−1^	301.75– 308.75 K	—	Self-recovery and wearability	[115]
Thermoelectric	PVA/betaine/I_2_/KI	Carbon papers	Sandwich structure	Temperature/pressure	0.65 mV K^−1^	298–313 K	~30 s	Dual-mode sensing and wearability	[116]
Potentiometric	PVA/Gly/NaCl	MXene/Al	Sandwich structure	Pressure/temperature	74.56 μA cm^−2^/%; 1.51 μA cm^−2^/°C	0–800 kPa; 5–75 °C	799 ms; 2.23 s	Broad detection range and dual-mode sensing	[127]
Potentiometric	PAAm/Gly/LiCl	V_2_O_5_/CNT	Porous structure	Pressure	51.7 kPa^−1^ (0–1.5 kPa), 39.7 kPa^−1^ (1.5–30 kPa), and 21.1 kPa^−1^ (30–500 kPa)	1.8 Pa– 1.5 MPa	~0.4 s	High sensitivity, broad response range, and capable of static detection	[128]
Triboelectric/piezoelectric	Gelatin/KCl	Au	Array	Pressure	—	<500 kPa	—	Decent dynamic tactile sensing performance	[131]
Triboelectric/potentiometric	PVA/NaCl /Gly	Al/PB@carbon	Micropatterned structure	Pressure	20 mV N^−1^, 17 mV N^−1^	0.01–10 N	~0.1 s	Detection and differentiation of both static and dynamic stimuli	[132]

## Applications

IHSSs enable sensory functions akin to human senses while also imitating the biological mechanism of ion migration. Its exceptional flexibility and sensitivity are further highlights of this technology [22]. Numerous hydrogel sensors with distinct self-powering mechanisms have been developed and widely used in domains like wearable electronics, environment monitoring, and HMI by utilizing the various energy harvesting technologies previously mentioned. These sensors not only eliminate the economic burden and environmental concerns associated with frequent battery replacements but also harness previously untapped clean energy. By doing so, they effectively mitigate the current energy crisis and reduce environmental pollution, making a substantial contribution to sustainable development. The promotion and application of this innovative technology will further drive scientific and technological progress, improving people’s quality of life.

### Wearable electronics

In recent years, self-powered sensors have drawn a lot of attention due to the ongoing advancements in wearable technology. Ionic hydrogels can adapt to a variety of wearable scenarios because of their excellent energy conversion performance, flexibility, compliance, and biocompatibility. IHSSs are wearable sensors that can be used to accurately sense physiological parameters like skin temperature, heart rate, pulse, and respiration rate in addition to monitoring large movements across different parts of the human body [38,202,203]. Additionally, self-powered sensors can be incorporated into smartwatches, smart apparel, and other gadgets to facilitate disease diagnosis and health monitoring. This allows users to access real-time health data to improve their management of their health [70,204,205].

**Fig. 16.**
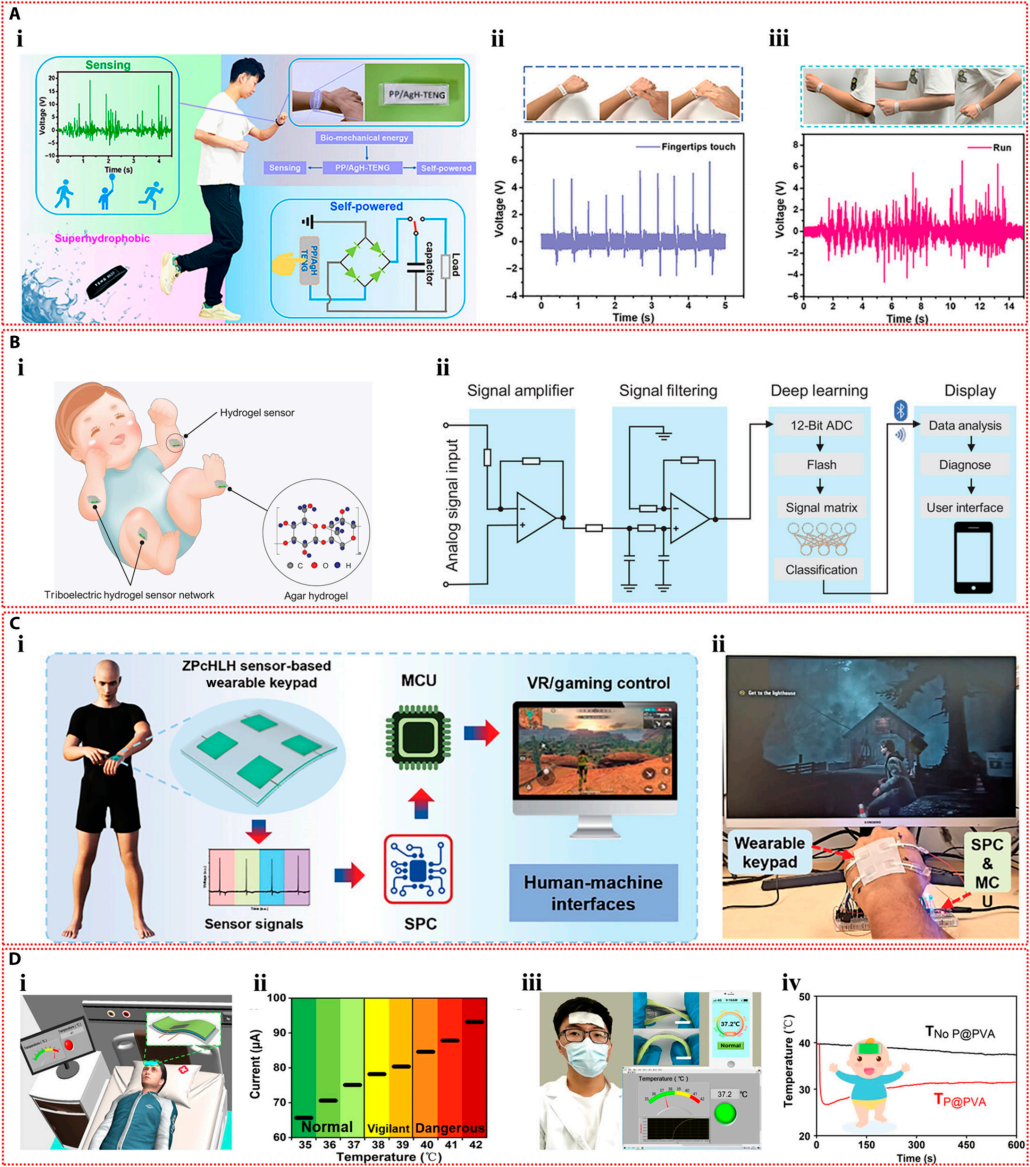
Applications for wearable electronics of IHSS devices. (A) Study of the wearable applications of the ionic hydrogel triboelectric sensor. (i) Self-powered sensing applications of a bracelet based on the ionic hydrogel triboelectric devices. Response signals generated by fingertip touching the bracelet (ii) and running (iii). Reproduced with permission from [206]. Copyright 2022, American Chemical Society. (B) Investigation of the wearable applications of the ionic hydrogel triboelectric sensor. (i) Schematic of the triboelectric sensor system for monitoring infant movement. (ii) Illustration of deep learning-assisted body area triboelectric sensor network for infant care system. Reproduced with permission from [207]. Copyright 2022, Wiley-VCH. (C) Study of the wearable applications of the ionic hydrogel triboelectric sensor. (i) Illustration of the system components for real-time control of the computer game from the wearable keyboard based on triboelectric sensors. (ii) Illustration of the wearable keyboard for game control. Reproduced with permission from [74]. Copyright 2023, Wiley-VCH. (D) Investigation of the wearable applications of the ionic hydrogel thermoelectric sensor. (i) Schematic of the ionic hydrogel thermoelectric patch for monitoring body temperature. (ii) Variations of current with body temperature in 3 representative areas. (iii) Schematic diagram of body temperature monitoring and the resulting temperature signal shown on the terminal. (iv) Cooling effect of the ionic hydrogel upon attachment to the skin surface. Reproduced with permission from [208]. Copyright 2021, American Chemical Society.

Qu et al. [206] prepared a superhydrophobic wearable triboelectric sensing wristband using a hydrogel composed of polyvinyl alcohol, sodium tetraborate, and silver nanowires as flexible electrodes, and polytetrafluoroethylene particles as the triboelectric layer (Fig. 16Ai). This self-powered sensing wristband could monitor human motion. When a fingertip touched the wristband dial, a corresponding output voltage was generated, waking up the dial (Fig. 16Aii). Additionally, the shaking of the wristband caused by wrist movement during running generated output signals due to the friction between the wristband and the wrist skin (Fig. 16Aiii). This self-powered sensor’s signal strength and frequency could be usefully employed to track different human motion states. This wristband showed a great deal of promise for wearable, self-powered sensing technology. Guo et al. [207] developed a triboelectric hydrogel sensor in a related advancement intended for thorough baby movement monitoring. This triboelectric sensor was composed of 3 layers: the water isolation layer was seaweed, the triboelectric layer was gelatin, and the electrode was an agar hydrogel with sodium chloride. A body area sensor network was created by carefully placing the self-powered sensor on various baby body parts to enable active monitoring (Fig. 16Bi). Furthermore, a baby care system that consists of an app display terminal, a deep learning algorithm system, and a signal processing system was proposed using this self-powered sensor network system (Fig. 16Bii). With the ability to identify patterns in a baby’s behavior and provide real-time remote monitoring, this sophisticated system shows great promise for intelligent wearable electronic devices.

Rahman et al. [74] combined LiCl electrolyte and nanofiller ZIF-8 into a poly(acrylamide)-co-hydroxyethyl acrylate hydrogel matrix to create a highly elastic and wear-resistant ionic hydrogel. Using this ionic hydrogel as an electrode, they fabricated a TENG. Wearable devices could use this TENG as an independent pressure sensor. Using a sensor array and modulation unit, a self-powered wearable keyboard system was created, which enables real-time command transmission to computer games (Fig. 16Ci). The computer game “Alan Wake” was effectively controlled by this wearable interface system (Fig. 16Cii), illustrating its usefulness in interactive gaming and wearable technology. Furthermore, in order to prepare an ionic hydrogel thermogalvanic generator, Bai et al. [208] introduced Fe^2+^/Fe^3+^ thermogalvanic couples into polyvinyl alcohol hydrogels and inserted a mesh-like polyvinylidene difluoride membrane into the gel to create a thermal barrier. The flexible gel thermoelectric patch was highly responsive to temperature changes and, when conformally attached to the human forehead, established a wearable self-powered body temperature monitoring system (Fig. 16Di). This system gradually increased its response with rising temperature, enabling precise monitoring of human body temperature (Fig. 16Dii). When the highly flexible gel patch was adhered to the forehead, receiving terminals including mobile phones and computers could display real-time human temperature (Fig. 16Diii). Additionally, the gel patch could provide a cooling effect to reduce fever in patients. The development of wearable medical electronics that run on their own energy has taken a new turn because of this design (Fig. 16Div).

### Human–machine interaction

The field of HMI has seen remarkable growth as a result of the emergence of artificial intelligence, which has encouraged HMI technologies to continue evolving and getting better [209,210]. Through HMI, users can communicate, operate, and interact with computer systems or other automated systems, leading to more efficient task completion and a richer user experience [211,212]. HMI research aims to design and develop user-friendly interfaces that meet user needs, enhance system usability, and improve user satisfaction [213]. With the development of technology, HMI has grown to be a fast-growing field of study that encompasses topics like augmented reality, virtual reality, and artificial intelligence. It is crucial for the advancement of technology going forward [214]. A critical foundation of HMI is flexible, wearable, and self-powered sensors. These sensors also have the ability to recognize physiological reactions or movements in humans and translate them into electronic signals that can be transmitted [215]. Through these sensors, systems can monitor users’ physiological states and behaviors in real time, providing accurate and instantaneous information feedback [216]. Self-powered sensing based on ionic hydrogels, due to its flexibility, high sensitivity, biocompatibility, and simple operating mechanisms, is particularly suitable for HMI applications and development.

**Fig. 17.**
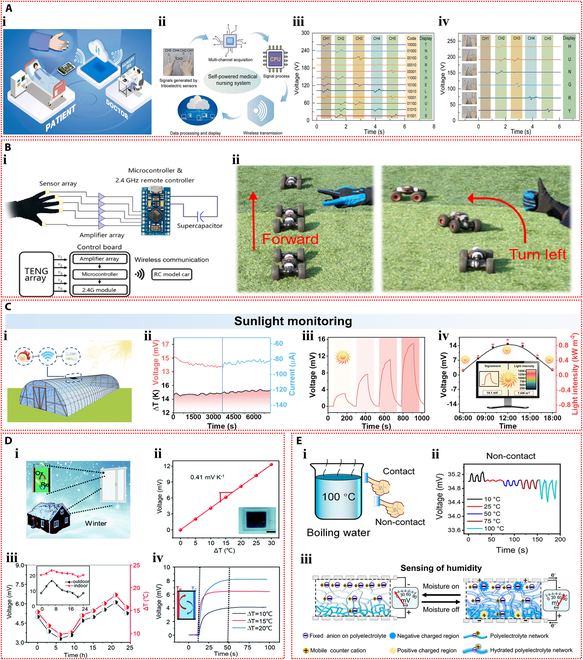
Applications for HMI of IHSS devices. (A) Investigation of the HMI applications of the ionic hydrogel triboelectric sensor. (i) Illustration of the self-powered medical care system used to remotely monitor and transmit patient requests for help. (ii) Illustration of the structural components of the self-powered medical care system. (iii) The collected signals from the 5 channels were encoded into the corresponding letters. (iv) Messaging of the word “hunger” achieved by the hybrid gestures. Reproduced with permission from [217]. Copyright 2022, Elsevier. (B) Study of the HMI applications of the ionic hydrogel triboelectric sensor. (i) Schematic of the structural composition of the communicator based on the ionic hydrogel triboelectric sensors. (ii) Photographs of the motions of a smart car controlled by different gestures. Reproduced with permission from [218]. Copyright 2021, MDPI. (C) Investigation of the environmental monitoring applications of the ionic hydrogel thermoelectric sensor. (i) Panoramic image of the ionic hydrogel window used for light intensity detection. (ii) Voltage and current responses of the thermoelectric sensor under temperature difference. (iii) The voltage response of the thermoelectric sensor increased with the intensity of the sunlight. (iv) Variations in voltage response of the thermoelectric sensor and the sunlight intensity over the whole day in summer. Reproduced with permission from [43]. Copyright 2024, Royal Society of Chemistry. (D) Study of the environmental monitoring applications of the ionic hydrogel thermoelectric sensor. (i) Illustration of the ionic hydrogel window used for outside temperature monitoring. (ii) Voltage variations of the ionic hydrogel window at different temperature differences. (iii) Variations in voltage and temperature difference of the ionic hydrogel window over a 24-h period. (iv) Response time of the ionic hydrogel window. Reproduced with permission from [219]. Copyright 2022, Royal Society of Chemistry. (E) Investigation of the environmental monitoring applications of the IHSS. (i) Diagram of the temperature sensing of the ionic skin affixed to the finger in contact and noncontact modes. (ii) Response signals generated in noncontact mode to heat source of different temperatures. (iii) Self-powered ionic skin for environmental humidity sensing. Reproduced with permission from [220]. Copyright 2022, American Chemical Society.

Using a flexible polyvinyl alcohol/phytic acid ionic hydrogel, Yang et al. [217] created a TENG that was used as a self-powered sensor in the HMI field for intelligent medical systems. The sensor, installed on the patient’s finger, generated signals as the finger bent to alert medical personnel (Fig. 17Ai). Figure 17Aii shows a schematic diagram of this medical monitoring system. The sensor produced electrical signals, which were gathered, handled, sent, and examined before being shown on a terminal screen. These signals were converted into matching letters after being recorded by a 5-channel data acquisition card, enabling the transfer of information via mixed gestures (Fig. 17Aiii and iv). In a related study, Zhang et al. [218] created a double-network ionic hydrogel that was utilized as the electrode in a triboelectric sensor. The hydrogel was made of polyacrylamide and sodium alginate that had been ion-crosslinked with sodium chloride. This triboelectric sensor was integrated into a smart glove and placed on the back of 5 fingers to serve as an autonomous gesture sensor. After that, the smart glove was incorporated into a self-powered HMI system, allowing for wireless remote control of a smart car’s movements (Fig. 17Bi and ii).

### Environment monitoring

The external environment’s temperature and humidity have a direct impact on how comfortable people feel in their surroundings. Therefore, monitoring and understanding changes in these parameters are crucial for improving living and working conditions. By monitoring and understanding changes in the external environment, corresponding measures can be taken to adjust and enhance comfort and health levels. The majority of temperature and humidity sensors that are currently on the market, however, need external power sources in order to operate, which makes their use difficult in a society that prioritizes reducing its carbon footprint. Ionic hydrogel-based flexible, self-powered sensors are becoming more and more important in environmental monitoring because they function without external power sources, supporting the objectives of sustainability and energy efficiency.

Yang et al. [43] prepared a polyvinyl alcohol-based organohydrogel containing Cu^2+^ and fabricated a light-controlled ionic organohydrogel thermoelectric sensor using copper oxide/copper foil as the electrodes. When exposed to sunlight, the electrodes facilitated photothermal conversion, creating a temperature difference in the ionic organohydrogel, thereby promoting the Cu/Cu^2+^ redox reaction to generate a thermoelectric output. This hydrogel thermoelectric sensor could serve as a light intensity monitor to track sunlight in greenhouses, preventing negative impacts on plant development due to excessive or insufficient light (Fig. 17Ci). The thermoelectric sensor could generate an output of 15.2 mV and −80 μA under a maximum temperature difference of 14.8 K (Fig. 17Cii). The output increased with increasing light intensity (Fig. 17Ciii). Figure 17Civ shows the sensor’s output signals throughout a summer day, which could be converted into real-time light intensity for solar monitoring in greenhouses. Moreover, Li et al. [219] introduced the I^−^/I_3_^−^ redox couple and glycerol into polyvinyl alcohol to prepare an antifreeze thermoelectric ionic hydrogel. An intelligent window constructed with this thermoelectric hydrogel sensor could accurately track the outdoor temperature in extreme environments, enabling self-powered temperature monitoring (Fig. 17Di). As the outside temperature dropped, the sensor’s output voltage increased and demonstrated strong consistency with variations in the outside temperature (Fig. 17Dii and iii). Furthermore, as shown in Fig. 17Div), this thermoelectric sensor demonstrated excellent self-powered temperature monitoring capabilities due to its quick response to temperature and ability to detect abnormal changes in the surrounding environment.

Xia et al. [220] utilized a hydrogel-assisted reaction–diffusion approach for manufacturing a gradient polyelectrolyte hydrogel self-powered sensor. The hydrogel network exhibited a gradient distribution of charged groups, capable of generating a self-induced potential in response to external temperature or humidity stimuli. Consequently, the ionic hydrogel performed admirably as an autonomous sensor, permitting accurate environmental temperature and humidity monitoring. The self-powered sensor could measure the temperature of heat sources in the surrounding air using both contact and noncontact techniques when it was attached to a finger (Fig. 17Ei and ii). By detecting the temperatures of the heat source, this sensor helped to avoid skin burns from coming into contact with overheated foreign objects. Furthermore, the sensor demonstrated high sensitivity to variations in environmental humidity, generating an induced potential with changes in humidity, thereby providing a reliable response to ambient humidity fluctuations (Fig. 17Eiii).

### Medical diagnostics

As one of the most promising flexible electronic materials, ionic hydrogels, which are flexible, stretchable, and biocompatible, can be directly connected to ionic devices or living tissues to transmit electrical signals, and have broad application prospects in the field of medical and health monitoring. Ionic hydrogel-based self-powered sensors that do not need to be powered by an external power source can be utilized to measure human physiological signals such as perspiration, respiration, blood pressure, and heart rate to assess the physiological state. Widely applied in a variety of biomedical applications, these sensors offer unique advantages for early health monitoring, ensuring direct, continuous, and highly accurate monitoring. The monitored physiological signals are of great importance for health management, disease prevention, and control.

**Fig. 18.**
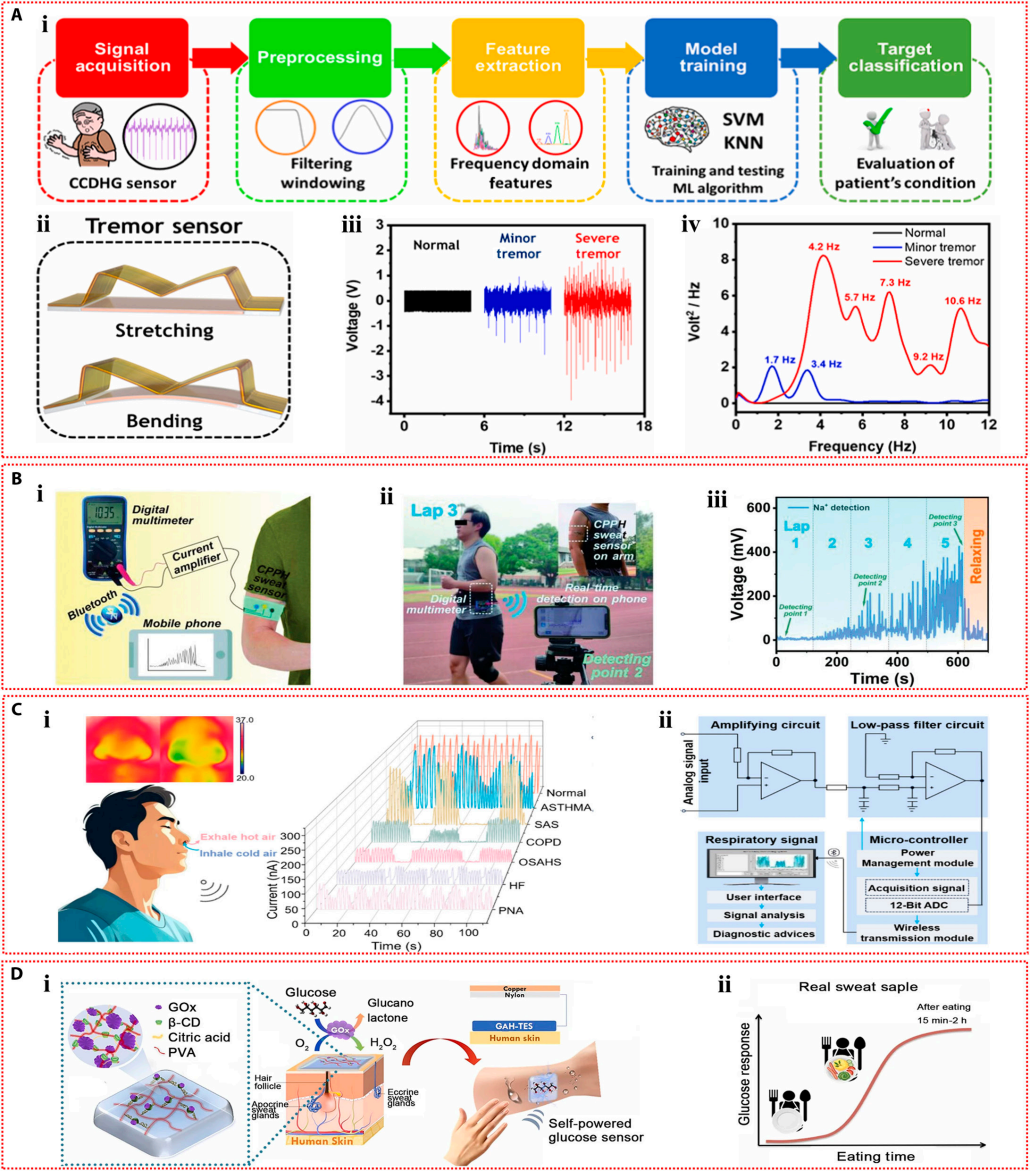
Applications for medical diagnostics of IHSS devices. (A) Study of the medical diagnostics of the ionic hydrogel triboelectric sensor. (i) Flow diagram for assessing the condition of a patient with Parkinson’s disease. (ii) Schematic image of the sensor while stretching and bending. (iii) Voltage signals of the sensor with 3 motions. (iv) Power spectral density of voltage signal from the sensor with 3 motions. Reproduced with permission from [79]. Copyright 2020, Elsevier. (B) Investigation of the medical diagnostics applications of the ionic hydrogel triboelectric sensor. (i) Schematic diagram of the connections between the sweat sensor and the wireless detection. (ii) Photos of the volunteer performing real-time wireless sensor test during the run. (iii) Real-time wireless sensing monitoring profiles of Na^+^ content in sweat. Reproduced with permission from [221]. Copyright 2022, Wiley-VCH. (C) Study of the medical diagnostics of the ionic hydrogel thermoelectric sensor. (i) Typical electrical signals of normal nose breathing and 6 respiratory disorders. (ii) Schematic diagram of the components of the respiratory monitoring strategy management circuit. Reproduced with permission from [222]. Copyright 2024, Elsevier. (D) Investigation of the medical diagnostics applications of the ionic hydrogel triboelectric sensor. (i) Schematic of the hydrogel sensor used to monitor glucose levels in sweat. (ii) Glucose levels in real sweat samples before and after meals measured by the sensor. Reproduced with permission from [223]. Copyright 2023, Elsevier.

For instance, Kim et al. [79] synthesized a highly stretchable ionic hydrogel based on catechol, chitosan, and diatoms. The ionic hydrogel can be employed as an electrode for the TENG. Furthermore, an M-shaped tremor sensor was developed based on the TENG to assess the low-frequency movements of Parkinson’s disease patients and combined with a machine learning algorithm to recognize the health status of patients (Fig. 18Ai and ii). The wrist-mounted tremor sensor generated clearly distinguishable voltage signals in response to different movements (Fig. 18Aiii). Moreover, the characteristics of hand tremor can be recognized by the power spectral density analysis (Fig. 18Aiv). This self-powered tremor sensor has great potential for application in disease prediction systems. Qin et al. [221] fabricated a rapidly self-healing, stretchable cellulose-based ionic hydrogel electrode. This electrode was encapsulated with PDMS and then assembled with an ion-selective membrane to fabricate a fully flexible self-powered sweat sensor capable of detecting real-time Na^+^, K^+^, and Ca^2+^ concentrations with high selectivity and sensitivity. Wirelessly transmitting the sensor output to the mobile phone application enables convenient real-time health monitoring (Fig. 18Bi and ii). The volunteer was made to wear the sensor while running, during which the device wirelessly monitored Na^+^ in sweat in real time, and the corresponding detection curve was displayed in Fig. 18Biii. The self-powered sweat sensor represents an innovative strategy for sensing sweat composition, which is feasible for potential applications in medical health monitoring.

Zhang et al. [222] presented a self-powered in-nostril sensor based on ionic thermoelectric hydrogel fiber by introducing an Fe^2+^/Fe^3+^ redox couple in polyvinyl alcohol, which enables long-term nonirritant anti-interference respiratory monitoring by identifying the temperature difference between the exhaled gas and skin inside the nasal cavity (Fig. 18Ci). Due to its slender structure and superior flexibility, the hydrogel fiber sensor can be easily inserted into the nasal cavity without causing any discomfort. Furthermore, a respiratory monitoring strategy management circuit was developed to accomplish the processing, transmission, and recognition of respiratory signals (Fig. 18Cii). With the assistance of deep learning, a respiratory monitoring strategy based on the self-powered sensor can actively identify multiple breathing patterns, offering a promising paradigm for bioelectronics-based early detection of respiratory diseases. Moreover, monitoring blood glucose levels in humans is critical for controlling diabetes, preventing complications, and assessing dietary health conditions. Kanokpaka et al. [223] introduced glucose oxidase encapsulated by β-cyclodextrin into an ionic hydrogel matrix composed of poly(vinyl alcohol) and citric acid, and then developed a self-healing glucose adaptive hydrogel-based triboelectric sensing sensor for blood glucose monitoring. The enzymatic reaction oxidizes glucose to produce gluconic acid and H_2_O_2_. The ionic strength increases when the glucose concentration in human sweat is elevated, leading to an increase in the conductivity of the hydrogel, which promotes the triboelectrification of the triboelectric system. The sensor realized self-powered continuous glucose monitoring and was highly selective and sensitive for measuring glucose concentration in human sweat before and after a meal (Fig. 18Di and ii), making it suitable for health monitoring of diabetic patients. In summary, IHSSs have a wide range of application prospects and important value in medical diagnosis. Through continuous technological innovation and optimization, it is believed that this technology will bring more breakthroughs and progress to the medical field in the future.

### Other applications

**Fig. 19.**
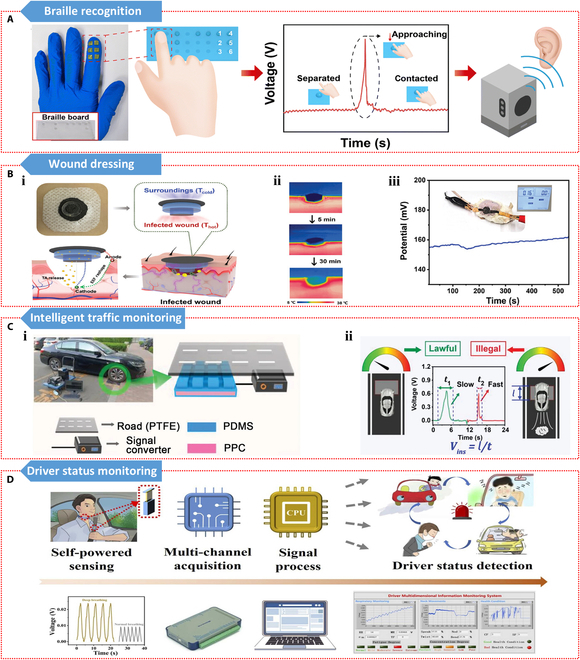
Other applications of IHSS devices. (A) Investigation of the braille recognition applications of the ionic hydrogel triboelectric sensor. Signals generated by an array of sensors attached to the index finger on touching the raised point of braille characters, which in turn converted into audio signals. Reproduced with permission from [224]. Copyright 2022, Royal Society of Chemistry. (B) Study of the wound dressing applications of the ionic hydrogel thermoelectric sensor. (i) Photograph of the device and schematic of the mechanism of EEF remodeling. (ii) Thermal images of the device on the wound. (iii) Real-time monitoring of the thermal potential produced by the device. Reproduced with permission from [225]. Copyright 2024, Wiley-VCH. (C) Investigation of the intelligent traffic monitoring applications of the ionic hydrogel triboelectric sensor. (i) Structure of the triboelectric sensor. (ii) Changes in the output signal of the device for monitoring the speed of the vehicle. Reproduced with permission from [226]. Copyright 2023, Wiley-VCH. (D) Study of the driver status monitoring applications of the ionic hydrogel triboelectric sensor. Flowchart of the driver multidimensional information monitoring system. Reproduced with permission from [73]. Copyright 2023, Elsevier.

Apart from the applications within the above 4 areas, IHSSs also have their unique applications in other specialized scenarios. For instance, braille recognition is a key component to smooth communication and learning for people with vision limitations. A self-powered braille sensing system that operates continuously and provides voice announcements can open up more diverse information channels for this group of people, thus improving their quality of life. Using polyacrylamide, clay, potassium iodide, and glycerol as raw materials, Dai et al. [224] prepared an ionic organohydrogel with self-healing and temperature resistance. A triboelectric braille recognition sensor was developed by utilizing the ionic organohydrogel as the electrode. When an array of sensors attached to a finger touched braille characters, the resulting electrical signals can be converted into corresponding audio signals for listening in real time (Fig. 19A). This work greatly facilitates communication and contact between the visually impaired and the outside world.

Hydrogel is the most competitive wound dressing candidate due to its good hydrophilicity and biocompatibility. Self-powered, flexible thermoelectric stimulation devices created by utilizing the temperature difference between the skin and the environment can be used as a green energy solution in the biomedical field. Self-powered flexible thermoelectric devices constructed by ionic thermoelectric hydrogels can be applied as novel wound dressings with great potential for wound treatment and monitoring. Gao et al. [225] developed an ionic thermoelectric hydrogel with excellent antimicrobial and antioxidant activities using acrylamide, 2-acrylamido-2-methyl-1-propanesulfonic acid sodium salt, and tannic acid. Self-powered flexible dressings constructed utilizing this thermoelectric hydrogel can provide a stable and sustained thermoelectric potential to remodel the endogenous electric field (EEF) of the wound while releasing tannic acid to act as an antiseptic and anti-inflammatory agent to accelerate wound healing (Fig. 19Bi and ii). After application to the wound, the hydrogel was monitored for temperature differences on the skin, which varied with contact time (Fig. 19Biii). The temperature difference between an infected wound and its surroundings causes a change in wound potential. The thermoelectric hydrogel can monitor the remodeled wound potential, and a stable remodeled EEF facilitates wound healing.

Moreover, IHSS also demonstrates its unique superiority in self-powered intelligent traffic monitoring systems. Li et al. [226] prepared an ionic hydrogel based on polyvinyl alcohol, sodium alginate, polyacrylamide, and tannic acid-modified cellulose nanocrystal. The ionic hydrogel encapsulated with PDMS was employed as the positive tribo-material and electrode, and polytetrafluoroethylene (PTFE) was utilized as the negative tribo-material (Fig. 19Ci). The developed triboelectric sensor can monitor the instantaneous speed and weight of the vehicle, which is important for effective traffic management (Fig. 19Cii). Besides, Luo et al. [73] constructed a triboelectric sensor based on ionic hydrogel containing NaCl and PVA. The triboelectric sensor was utilized to design a multidimensional information monitoring system for drivers based on smart neck ring and seat belt (Fig. 19D). By monitoring the neck movement and chest breathing of the driver in real time, the system can obtain detailed information about the health, fatigue, and concentration level of the driver. This research is of vital importance to deepen the development and progress of driver condition monitoring technology.

## Conclusion and Perspectives

**Fig. 20.**
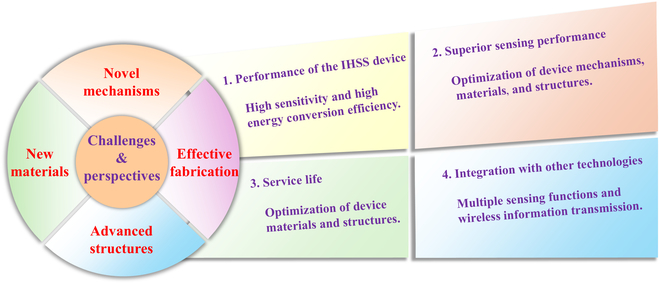
Future development trends of IHSSs.

Due to its flexibility, high sensitivity, design flexibility, self-healing ability, and biocompatibility, ionic hydrogels have become a very promising material for realizing flexible self-powered sensing devices. Ionic hydrogels are used as sensing materials in self-powered sensors, and this paper reviews the research progress in this area. The focus is on providing an overview of the various energy conversion processes used in self-powered sensing systems made of ionic hydrogels. Depending on the specific application scenario, sensors must be equipped with the ability to generate their own energy by harvesting renewable energy from the surrounding environment or the human body. Additionally, high-performance self-powered sensors can be realized by carefully designing the structural framework and optimizing the performance of ionic hydrogels. Typical applications of IHSS devices in wearable electronics, HMI, environment monitoring, and medical diagnostics are also discussed. Despite the substantial advancements made in IHSS devices in recent years, they are in the early stages of research, many challenges still need to be overcome, and there is room for the development of new technologies (Fig. 20).

The main focus of upcoming research and development should be on improving the sensitivity and energy conversion efficiency of IHSS devices. The improvement is crucial for advancing their performance and expanding their practical applications. Currently, the energy conversion efficiency in IHSS devices is relatively low, which hampers the full utilization of environmental energy sources, resulting in short operation time and poor stability. For the application of ionic hydrogels as electrodes in triboelectric sensors, optimizing electrode design, material selection, and surface treatment can enhance sensor sensitivity and energy harvesting efficiency. The molecular and structural design of ionic hydrogels should be the main focus of efforts for self-powered sensors that use thermoelectric, piezoelectric, and ion diode mechanisms. This will increase the concentration gradient differences and improve ion directional transport. Self-powered sensors will function and operate more efficiently as a result of these advancements. It is crucial to take into account the Faradaic reactions that take place at the interface between the ionic hydrogel and the electrodes when dealing with potentiometric sensors. Furthermore, the development of new energy conversion mechanisms and superior sensing performance is one of the main directions for the future advancement of IHSS devices. This requires an adequate combination of novel mechanisms, new materials, advanced structures, and effective fabrication methods.

Moreover, the reliance on a single energy harvesting source poses a challenge for current IHSS applications. The design of hybrid energy harvesting technologies offers a promising solution. Future IHSS will not be limited to single sensing functions but will integrate multiple sensing functions, such as monitoring temperature, pressure, humidity, and light, to meet the demands of various fields. Achieving the capability to utilize different sensing principles simultaneously to detect and distinguish complex external stimuli, similar to the functionality of human skin, is highly anticipated. Additionally, incorporating machine learning methods to analyze and process various types of sensor data can facilitate intelligent decision-making and feedback. This approach can equip IHSS devices with multimodal autonomous sensing capabilities, further enhancing their functionality and adaptability in diverse applications.

The stability and durability of materials are vital for ensuring the performance and longevity of sensors. Ionic hydrogel materials may experience issues such as swelling, shrinking, or aging over prolonged use, which need to be addressed to enhance the reliability and lifespan of the sensors. Crosslink density is a key factor affecting the swelling performance of hydrogels. Hydrogels with anti-swelling performance can be fabricated by adjusting the cross-linking density. The anti-swelling performance can be enhanced by adjusting the amount of cross-linking agent or by introducing special ingredients to form strong electrostatic interactions or hydrogen bonding interactions within the hydrogel. Furthermore, ionic hydrogels are inevitably subject to shrinkage or aging due to water loss when used at room temperature for a long period of time or at high temperatures. The organic solvents can be introduced into the hydrogels to avoid water loss as much as possible. Also, the entire device can be encapsulated to prevent water loss and aging of the hydrogel material. Thus, advanced encapsulation techniques should be further developed to minimize the impact of the external environment on IHSS. The lifetime of IHSS devices is one of the crucial factors affecting their performance. IHSS based on pressure response are subject to deformation during operation. On the one hand, physical factors that affect the lifetime of IHSS include material fatigue, cracking, and wear. On the other hand, chemical factors affecting the lifetime of a portion of the IHSS include ion concentration polarization, irreversible redox reactions, and ion pathway alterations. Hence, these influences should be considered in the future design to improve the stability and durability.

Although ionic hydrogels are flexible and compliant, making them ideal for wearable applications, most studies of IHSS have relied on wired connections. The presence of multiple exposed wires is not suitable for wearable applications and restricts their use in commercial electronic devices. To enhance the convenience and functionality of hydrogel devices, future applications of IHSS should focus on integrating wireless information transmission technologies, enabling wireless capabilities and broader adoption.

It is expected that the development and synthesis of new ionic hydrogel materials will enhance sensor functionality and performance, given the ongoing progress in materials science and nanotechnology. Additionally, the integration with other sensing technologies will bring more application opportunities and innovative breakthroughs for IHSS. With continuous advancements in science and technology, IHSSs are expected to introduce greater convenience and innovation to human life and health.

## Data Availability

The data that support the findings of this study are available from the corresponding authors upon reasonable request.
